# *Trichoderma* and its role in biological control of plant fungal and nematode disease

**DOI:** 10.3389/fmicb.2023.1160551

**Published:** 2023-05-03

**Authors:** Xin Yao, Hailin Guo, Kaixuan Zhang, Mengyu Zhao, Jingjun Ruan, Jie Chen

**Affiliations:** ^1^College of Agronomy, Guizhou University, Guiyang, China; ^2^Science and Technology Innovation Development Center of Bijie City, Bijie, China; ^3^Institute of Crop Science, Chinese Academy of Agriculture Science, Beijing, China; ^4^School of Agriculture and Biology, Shanghai Jiao Tong University, Shanghai, China

**Keywords:** *Trichoderma*, plant diseases, biological control, growth promotion, action mechanism

## Abstract

*Trichoderma* is mainly used to control soil-borne diseases as well as some leaf and panicle diseases of various plants. *Trichoderma* can not only prevent diseases but also promotes plant growth, improves nutrient utilization efficiency, enhances plant resistance, and improves agrochemical pollution environment. *Trichoderma* spp. also behaves as a safe, low-cost, effective, eco-friendly biocontrol agent for different crop species. In this study, we introduced the biological control mechanism of *Trichoderma* in plant fungal and nematode disease, including competition, antibiosis, antagonism, and mycoparasitism, as well as the mechanism of promoting plant growth and inducing plant systemic resistance between *Trichoderma* and plants, and expounded on the application and control effects of *Trichoderma* in the control of various plant fungal and nematode diseases. From an applicative point of view, establishing a diversified application technology for *Trichoderma* is an important development direction for its role in the sustainable development of agriculture.

## Introduction

In the traditional crop cultivation process, the excessive use of pesticides and chemical fertilizers, as well as the long-term large-scale planting of a single crop, has led to the destruction of the farmland ecological environment, plant diseases, insect pest problems, crop pesticide residues, and soil and water environment pollution (Bardin et al., 2015). With green agricultural development, people are urgently seeking safe, effective, and environmentally friendly plant disease control measures. Biological control is mainly used to control harmful organisms in plants through beneficial organisms and their products to control plant diseases and effectively reduce the application of chemical fertilizers and pesticides (Harman et al., 2021). *Trichoderma*, a biological fungus widely used for plant pest control, mainly exists in the soil, air, plant surface, and other ecological environments and can effectively control a variety of plant diseases (Haouhach et al., 2020; Zheng et al., 2021; Wang R. et al., 2022). *Trichoderma* is mainly used to control soil-borne diseases in various plants and some leaf and spike diseases (Samuels et al., 2006; Vicente et al., 2020; Abbas et al., 2022). *Trichoderma* can prevent disease, promote plant growth, improve nutrient utilization efficiency, enhance plant resistance, and repair agrochemical pollution (Tilocca et al., 2020; Fontana et al., 2021; Sánchez-Montesinos et al., 2021; Al-Surhanee, 2022; Tyśkiewicz et al., 2022).

*Trichoderma* belonging to *Eumycota*, *Deuteromycotina*, *Hyphomycetes*, *Hyphomycetales*, and *Moniliaceae* (Kubicek et al., 2019). Its sexual stage includes the *Ascomycota*, Sordariomycetes, *Hypocreales*, *Hypocreaceae,* and *Trichoderma* ssp. (Sun et al., 2012). There are more than 370 *Trichoderma* spp. including *T. harzianum*, *T. viride*, *T. asperellum*, *T. hamatum*, *T. atroviride*, *T. koningii, T. longibrachiatum*, and *T. aureoviride* (Sánchez-Montesinos et al., 2021; Sun et al., 2022). *Trichoderma* has been used in biological control research, including *T. harzianum*, *T. hamatum*, *T. longibrachiatum*, *T. koningii*, *T. viride*, *T. polysporum*, and *T. asperellum* (Di Marco et al., 2022). Many studies have shown that most *Trichoderma* spp. can produce bioactive substances and have antagonistic effects on plant-pathogenic fungi and plant-pathogenic nematodes (Druzhinina et al., 2018). These bioactive substances, including secondary metabolites and cell wall-degrading enzymes, can effectively improve crop resistance, reduce plant diseases, and promote plant growth (Kubicek et al., 2019). Professor Harman of Cornell University isolated and purified *T. harzianum* T22 strain and systematically studied its application in biological control of plant pests and commercial development of biological control technology (Harman, 2000). This study systematically and comprehensively elaborated on the research progress on *Trichoderma* spp. and its role in plant disease control, its application as a biological control and its mechanism, as well as preliminarily discussed the problems and prospects of *Trichoderma* as a biological control agent, providing a reference for future research and application.

## Application and mechanism of action of *Trichoderma* in plant fungal disease control

### Application of *Trichoderma* in biological control of plant fungal diseases

*Trichoderma* is a biocontrol fungus widely distributed worldwide. *Trichoderma* has a huge application value and potential in the field of biological control of plant diseases (Tyśkiewicz et al., 2022). Research on the use of *Trichoderma* to control plant diseases has been reported worldwide. *T. viride* and *T. harzianum* have different degrees of inhibitory effects on 29 species of plant pathogenic fungi belonging to 18 genera, including *Botrytis*, *Fusarium*, and *Rhizoctonia*. *Trichoderma* has control effects on a variety of plant pathogenic fungi, such as *Rhizoctonia solani*, *Pythium ultimum*, *Fusarium oxysporum*, *Sclerotinia sclerotiorum*, *Botrytis cinerea, Pseudocercospora* spp. and *Colletotrichum* spp. (Tian et al., 2016, 2018; Saravanakumar et al., 2017; Debbi et al., 2018; Li et al., 2018; Bubici et al., 2019; Filizola et al., 2019; Herrera-Téllez et al., 2019; Álvarez-García et al., 2020; Andrade-Hoyos et al., 2020; Carro-Huerga et al., 2020; Damodaran et al., 2020; Zhang et al., 2020, 2021; Al-Askar et al., 2021; Chen et al., 2021; Degani and Dor, 2021; Dugassa et al., 2021; Intana et al., 2021; Zhang C. et al., 2022; Zhang Y. et al., 2022). *Trichoderma* has been widely used for the biological control of cotton verticillium wilt, crop gray mold, tomato gray mold, melon wilt, potato dry rot, tobacco root rot, and other plant diseases (Rashmi et al., 2016; Andrade-Hoyos et al., 2020; Alfiky and Weisskopf, 2021; Lazazzara et al., 2021; Leal et al., 2021; Manganiello et al., 2021; Degani et al., 2021a; Pollard-Flamand et al., 2022; Rees et al., 2022; Risoli et al., 2022). *T. longibrachiatum* T6 biocontrol agent has a good control effect on pepper damping off and can effectively control the spread of pepper disease (Girma, 2022). The control effect was up to 54.8%, which is 12.5% higher than that of the chemical pesticide carbendazim (Yuan et al., 2019; Al-Askar et al., 2022)*. T. harzianum* has a good control effect on pepper and potato *Phytophthora* blight. It can inhibit the growth of *Phytophthora* blight in soil, reduce the number of pathogenic fungi, and effectively reduce the rate of dead seedlings and disease index of plants (Guzmán-Guzmán et al., 2017; Kappel et al., 2020; Mahmoud et al., 2021; Liu Y. et al., 2022). The control effect of 50× *T. asperellum* fungal fluid on apple canker reached 88.24%, which was significantly higher than that of benziothiazolinone (Ruangwong et al., 2021a). *T. asperellum* has different effects on different pathogenic fungi, among which its inhibitory effect on the pathogen causing corn leaf spot is the best, at up to 77.91%, followed by *Pythium* and *Fusarium*; and the worst inhibition effect is on corn sheath blight (Guo et al., 2019; Intana et al., 2022). Therefore, using *Trichoderma* to prevent and control plant diseases can not only inhibit the growth of pathogenic fungi, which is conducive to plant growth but can also reduce the use of chemical pesticides, which is conducive to protecting the ecological environment.

### Storage resistance and processing technology of *Trichoderma* products

The commercial application of biocontrol *Trichoderma* depends to a large extent on the stress resistance (such as high temperature, drying, ultraviolet radiation, etc.) and storage resistance (more than 1 year at normal temperature) of the *Trichoderma* preparation (Alfiky and Weisskopf, 2021). At present, there are two main technologies: on the one hand, reducing acidity and regulating oxygen utilization to induce *Trichoderma* to produce stress-resistant chlamydospores; on the other hand, some chemical additives (such as copper) are added to the preparation. Monfil and Casas-Flores (2014) increased the resistance of *Trichoderma* to high temperature (35 ~ 40°C) and ultraviolet radiation by adding trehalose to *Trichoderma*. Monfil and Casas-Flores (2014) added glycerin to the *Trichoderma* preparation as a humectant to prolong its shelf life. Special packaging design, vacuum drying, and low-density polyethylene packaging materials can extend the shelf-life to 15 months. In the field of *Trichoderma* preparation form processing, Chen et al. (2021) developed the *Trichoderma* conidia powder agent and obtained a patented technology for inducing *Trichoderma* to produce chlamydospores. With the increasingly mature biological control technology, the types of commercial preparations for *Trichoderma* spp. are also becoming diverse. There are four main categories: (1) Wettable powders, which are made by mixing conidia powder, powdery carriers, and humectant. (2) Granules are made by mixing and stirring conidia and carrier. (3) A mixture consisting of spore powder and chemical fungicides mixed in proportion on a suitable carrier. (4) Suspenso-emulsion is prepared by suspending conidia in a lotion composed of vegetable oil, mineral oil, emulsifier, etc. In the current market for *Trichoderma* biological agents, *T. Harzianum* is the largest, followed by *T. viride* and *T. koningii*. *Trichoderma* agents widely used in plant disease control mainly include Trichodex (Makhteshim Chemical Works Ltd., Israel), a commercial preparation of *T. harzianum* T-39; RootShield (Bioworks, USA), a commercial preparation of *T. harzianum* T-22; Binab TF (Binab Bio Innovation AB, Sweden), a mixed-agent of *T. harzianum* and *T. polyspora*; Sentinel (Novozymes, Denmark), a commercial preparation of *T. atrovilide*; And Supervivit (Borregaard Bioplant, Denmark), a commercial preparation of *T. harzianum*.

### Mechanism of *Trichoderma*-induced endophytic microbiome synergistically stimulating plant immune response

#### Competitive role of *Trichoderma*

**Figure 1:**
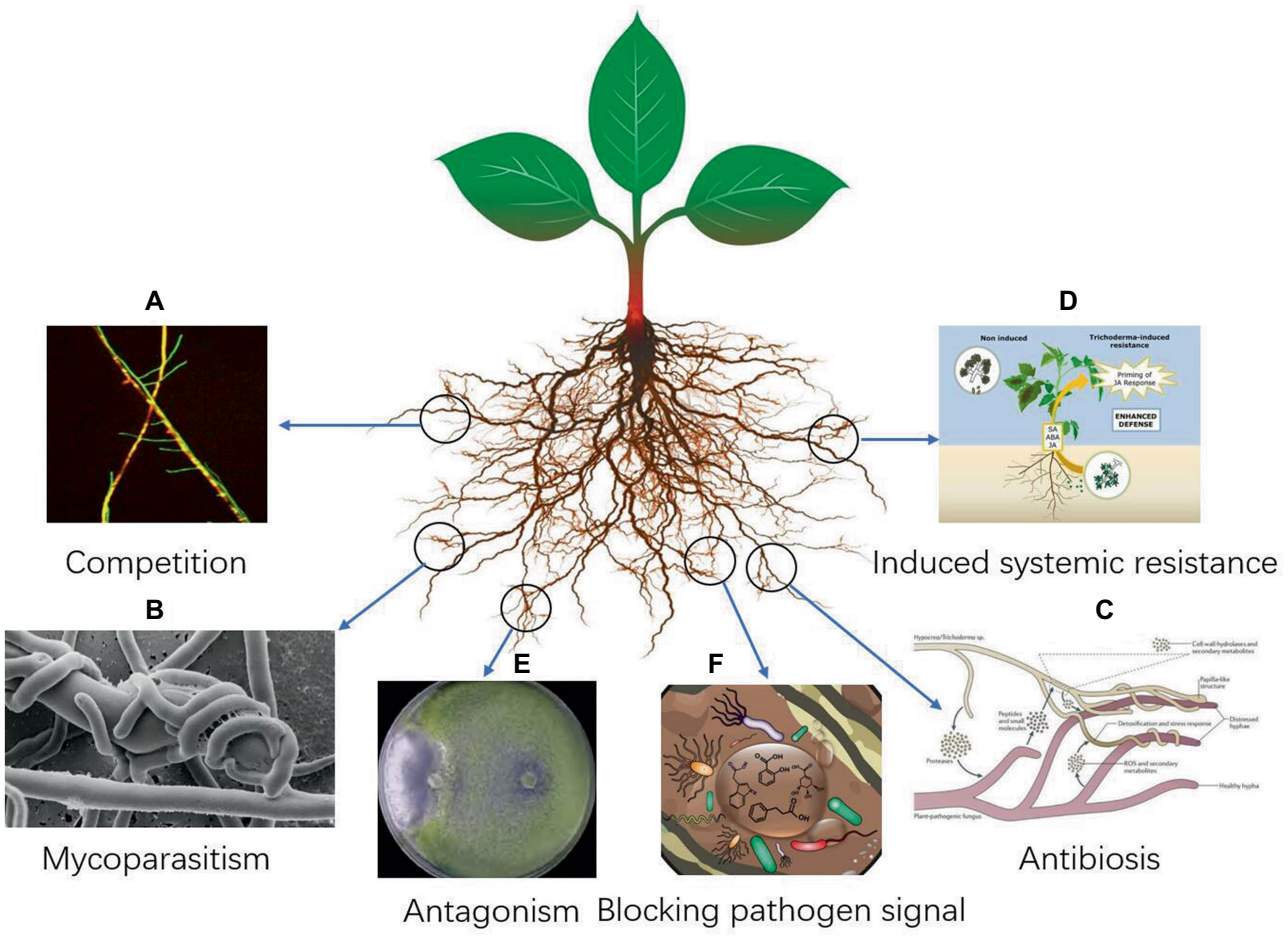
**(A–F)** Schematic diagram of the mechanism of action of *Trichoderma* in plant fungal disease control (Martínez-Medina et al., 2013; Lamdan et al., 2015; Morán-Diez et al., 2020; Manzar et al., 2022; Tamizi et al., 2022).

*Trichoderma* are saprophytic fungi with fast mycelial growth and strong adaptability to the environment. It can seize the invasive part of the pathogenic fungi in the root of a plant, thus hindering the invasion of the pathogen fungi. It can also rapidly absorb the nutrients required for the growth of the pathogen fungi, resulting in nutrient deficiency and inhibiting the growth and reproduction of the pathogen fungi (Guo et al., 2019; Bazghaleh et al., 2020; Halifu et al., 2020; Figure 1A). *Trichoderma* has strong adaptability to the environment (Köhl et al., 2019; Morán-Diez et al., 2019; Pescador et al., 2022; Xu et al., 2022). Through its rapid growth and reproduction, it can seize nutrients and space near the plant rhizosphere, consume oxygen in the air, and weaken the growth of plant pathogenic fungi (Basińska-Barczak et al., 2020; Oszust et al., 2020; Panchalingam et al., 2022). The growth rate of *Trichoderma* is much faster than that of plant-pathogenic fungi; therefore, it can effectively inhibit the growth of plant-pathogenic fungi (Mohiddin et al., 2021). After entering the soil for 24 h, *Trichoderma* can quickly adsorb to the roots of crops for propagation, and the hyphae quickly wrap the roots of crops to form a protective layer, protect the roots of crops from the invasion of pathogens, and kill the nearby pathogens. Risoli et al. (2022) found that the growth rate of *T. harzianum* was 2.0 to 4.2 times faster than that of *B. cinerea*. *Trichoderma* mycelium competed with *Fusarium graminearum* by clinging, twining, inter-penetration, and other mechanisms, which caused the mycelium of *F. graminearum* to deform and eventually disappear (Dugassa et al., 2021). *Trichoderma* can capture water and nutrients, occupy space, and consume oxygen, etc. through rapid growth and reproduction, to weaken and exclude the gray mold pathogen in the same habitat (Herrera-Téllez et al., 2019).

#### Mycoparasitism of *Trichoderma*

Mycoparasitism is one of the important mechanisms in the biological control of *Trichoderma* (Figure 1B). *Trichoderma* can parasitize about 18 genera of *Pythium*, *Phytophthora*, *Rhizoctonia*, and *Peronospora*. They directly invade or wound the mycelium, causing the pathogen cells to expand, deform, shorten, become round, shrink the protoplasm, and break the cell wall. *Trichoderma* TM can invade the hyphae of *Sclerotinia sclerotiorum*, attach to and wrap around the hyphae of pathogenic fungi, and break the hyphae of *S. sclerotiorum* until it disintegrates (Shaw et al., 2016). Risoli et al. (2022) found that *Trichoderma* can form putrescence in a specific environment, which has a mycoparasitic effect on *Botrytis cinerea*. It forms a large number of branches and sexual structures after entering the host hyphae, thus inhibiting the appearance of grape *B. cinerea* symptoms (Aswani et al., 2022). *Trichoderma* can degrade the cell wall of pathogenic fungi by secreting chitin-degrading enzymes, so as to better invade the interior of pathogenic fungi. *Trichoderma* mycelium hyperparasitized *Fusarium graminearum* by clinging, twining, inter-penetration, and other mechanisms, which caused the mycelium of *F. graminearum* to deform and eventually disappear (Tian et al., 2018). Chitinase secreted by *T. harzianum* plays an important role in promoting cell wall dissolution, mycelial autolysis, chitin assimilation, fungal parasitism, and inhibiting spore germination, mycelial growth, and spore formation (Saravanakumar et al., 2017). *T. koningiopsis* can invade the hyphae of *Sclerotinia sclerotiorum*, attach to and wrap around the hyphae of pathogenic fungi, and break the hyphae of *S. sclerotiorum* until it disintegrates (Shaw et al., 2016).

#### Antibiosis effect of *Trichoderma*

Antibiosis mainly refers to the ability of *Trichoderma* to inhibit the growth of plant pathogenic fungi by secreting antagonistic substances (Kottb et al., 2015; Izquierdo-García et al., 2020; Morán-Diez et al., 2020; Shobha et al., 2020; El-Hasan et al., 2022; Figure 1C). *Trichoderma* can produce hundreds of antimicrobial secondary metabolites, including trichomycin, gelatinomycin, chlorotrichomycin, and antibacterial peptides (Maruyama et al., 2020). These secondary metabolites can act as antibacterial agents, promote plant growth, and provide rich materials for the development of agricultural antibiotics (Nawrocka et al., 2018). Naglot et al. (2015) found that the metabolites of *T. viride* had a significant inhibitory effect on the wilt-specific form of *F. oxysporum*, with an inhibition rate of 54.81%. Manganiello et al. (2018) found that the volatile secondary metabolites secreted by *T. viride* TG050 609 can cause the mycelium of *P. nicotianae* to grow irregularly, break, or even dissolve, proving that *T. viride* has an antibiosis effect on *P. nicotianae*. In addition, most *Trichoderma* strains can produce antimicrobial substances such as pentaibols, which can inhibit a variety of plant pathogenic fungi and can also cooperate with cell wall-degrading enzymes on pathogenic fungi to effectively inhibit their growth (Debode et al., 2018; Mayo-Prieto et al., 2019; Kovács et al., 2021; Martínez-Salgado et al., 2021; Tamizi et al., 2022). Some studies have shown that some *Trichoderma* spp. can produce volatile metabolites, which can inhibit the growth of colonies to varying degrees, and some of them can inhibit the growth of colonies by more than 80% (Navazio et al., 2007; Vos et al., 2015; Samuelian et al., 2016; Marik et al., 2019; Thambugala et al., 2020; Kong et al., 2022; Li M. et al., 2022).

In recent years, research on the genome, transcriptome, proteome, and metabolome of *Trichoderma* has developed rapidly (Zhang Y. et al., 2022). Genome and EST sequencing, and microarray and microarray based expression profiling have become important tools for exploring *Trichoderma* genes and studying the mechanism of action (Tamizi et al., 2022). In genomics research, a cDNA library of *T. harzianum* EST has been constructed, and multiple new genes have been identified (Ferreira Filho et al., 2020). The researchers completed the genome sequencing of *T. reesei*, *T. virens*, and *T. atroviride*. Rubio et al. (2014) used high-density oligonucleotide (HDO) microarray technology and bioinformatics analysis to detect and analyze: after 20 h of interaction between T. hamatum T7 and tomato, there were 200 differentially expressed genes, of which 166 were up-regulated and 34 were down-regulated; 43.14% of genes are related to molecular function, 56.86% are related to biological processes, and 32.0% are related to cell component formation. Shoresh and Harman (2010) identified the changes of 27 endochitinase genes and 4 exochitinase genes in maize after interaction between *T. harzianum* T22 and maize using proteomic methods and EST libraries and discovered a new specific chitinase. Chen et al. (2021) used proteomic techniques to identify proteins related to resistance to root rot in maize, among which chitinase, SOD, isoflavone reductase, and PR protein are associated with resistance to root rot in maize seedlings.

#### Induced systemic resistance of *Trichoderma*

*Trichoderma* can induce host plants to produce defense responses. While inhibiting the growth and reproduction of pathogenic fungi, it can also induce crops to produce self-defense systems to obtain local or systemic disease resistance (Figure 1D). *Trichoderma*-induced plant disease resistance is achieved through two approaches: one is to regulate the plant disease resistance response by regulating elicitors or effectors; second, the cell wall-degrading enzyme produced by *Trichoderma* releases oligosaccharides that can induce plant resistance (Gomes et al., 2015). At present, there are more than 10 elicitors of *Trichoderma* that induce plant resistance, including Sm1, QID74 hydrophobic protein, chitin-degrading enzyme, MRSP1, xylanase, cellulase, endopolygalacturonase, sucrase, and antibacterial peptides. These substances are mainly derived from five *Trichoderma* species: *T. asperellum*, *T. viride*, *T. atroviride*, and *T. harzianum* (Karimi Aghcheh et al., 2013; Lamdan et al., 2015; Ngo et al., 2021; Matas-Baca et al., 2022; Zaid et al., 2022; Zhu et al., 2022). Saravanakumar et al. (2016) found that the activities of peroxidase (POD) and phenylalanine ammonia lyase (PAL) of corn seeds coated with *Trichoderma* increased significantly, and the plants were resistant to curvularia leaf spot of corn.

#### Antagonism of *Trichoderma*

The antagonism of *Trichoderma* is often considered the result of simultaneous or sequential action of more than two mechanisms (Saravanakumar et al., 2016; Sui et al., 2022; Figures 1E,F). Based on multiple mechanisms, *Trichoderma* has synergistic capabilities (Alonso-Ramírez et al., 2014; Moreno-Ruiz et al., 2020; Stracquadanio et al., 2020; Alukumbura et al., 2022; Chung et al., 2022; Kappel et al., 2022). Jogaiah et al. (2018) found that the synergistic use of *T. harzianum* and fungicides can effectively inhibit tomato gray mold, and the inhibition rate was higher than that of both fungicides alone. Zhang et al. (2017) found that the fermentation metabolites of *T. viride* CCTCC-SWB0199 and brassinolide in a certain proportion had a higher effect on the control of tomato gray mold than when the two were applied separately. Jogaiah et al. (2018) found that the biocontrol effect of *Trichoderma* spp. against plant pathogens fungi are often the result of a combination of multiple mechanisms, and different strains have different emphasis on biocontrol mechanisms. Monfil and Casas-Flores (2014) used transcriptology and metabolomics to study the tripartite interactions of *Arabidopsis*, *Trichoderma*, and *Pseudomonas syringae* tomato varieties. The results showed that the treatment of *Arabidopsis* roots with *Trichoderma* for 48 h induced more than 300 gene expression changes in the roots, but the changes in leaf genes were different from those in the roots (Monfil and Casas-Flores, 2014). *Trichoderma* induces the differential expression of host plant genes, mainly at the level of quantity (Viterbo et al., 2005; Malmierca et al., 2012; Park et al., 2019). A metabolomics study found that 27 compounds were related to induced resistance in *Arabidopsis thaliana* (Monfil and Casas-Flores, 2014). The biocontrol effect of *Trichoderma* on plant pathogenic fungi is often the result of multiple mechanisms, and different strains have different biocontrol mechanisms (De Zotti et al., 2020; Cai et al., 2021; Ji et al., 2021; Ruangwong et al., 2021b; Figure 1).

## Application and mechanism of action of *Trichoderma* in plant nematode disease control

### Application of *Trichoderma* in the control of plant nematodes

At present, the reported *Trichoderma* with nematicidal activity mainly includes *T. longibrachiatum*, *T. viride*, *T. harzianum*, *T. Hamatum*, *T. atroviride,* and *T. koningii* (Zhu et al., 2022). The fermentation broth of *T. longibrachiatum* T6 has a strong lethal effect on the eggs and second-instar larvae of cereal cyst nematodes in wheat (Zhu et al., 2022). The relative inhibition rate of the two concentrated fermentation broths on egg hatching was 46.47%, and the corrected mortality rate for the second-instar larvae was 44.45% (Sokhandani et al., 2016). Microscopic observation showed that the fermentation liquid of *T. longibrachiatum* T6 could digest the contents of nematode eggs and body cavities of the second instar larvae (Zhu et al., 2022). Khan et al. (2020) used inducers to make *T. koningiopsis* UFSMQ40 produced fermentation broth containing a large amount of chitinase, and its lethal rate to root-knot nematodes of South China and Java was 90.4 and 63.2%, respectively. Baazeem et al. (2021) analyzed the transcriptional activity of chi18-5 and chi18-12 genes of *T. harzianum* FB10 in *Trichoderma* egg parasitism. Compared to the control, the expression of chi18-5 and chi18-12 genes during parasitism increased significantly, indicating that the chitinase content increased, which could provide favorable conditions for egg cleavage (Baazeem et al., 2021).

### Mechanism of *Trichoderma* resistance to nematode disease

The mechanism by which *Trichoderma* inhibits nematode disease remains unclear. Some studies suggest that the serine protease pr1 of *Trichoderma* has similar biochemical characteristics to the protein Pr1 of nematicidal fungi, so it has a certain nematode inhibition effect (Forghani and Hajihassani, 2020). The antimicrobial peptides produced by *Trichoderma* also have nematicidal effects (Fan et al., 2020).

### Mycoparasitism effect of *Trichoderma*

**Figure 2:**
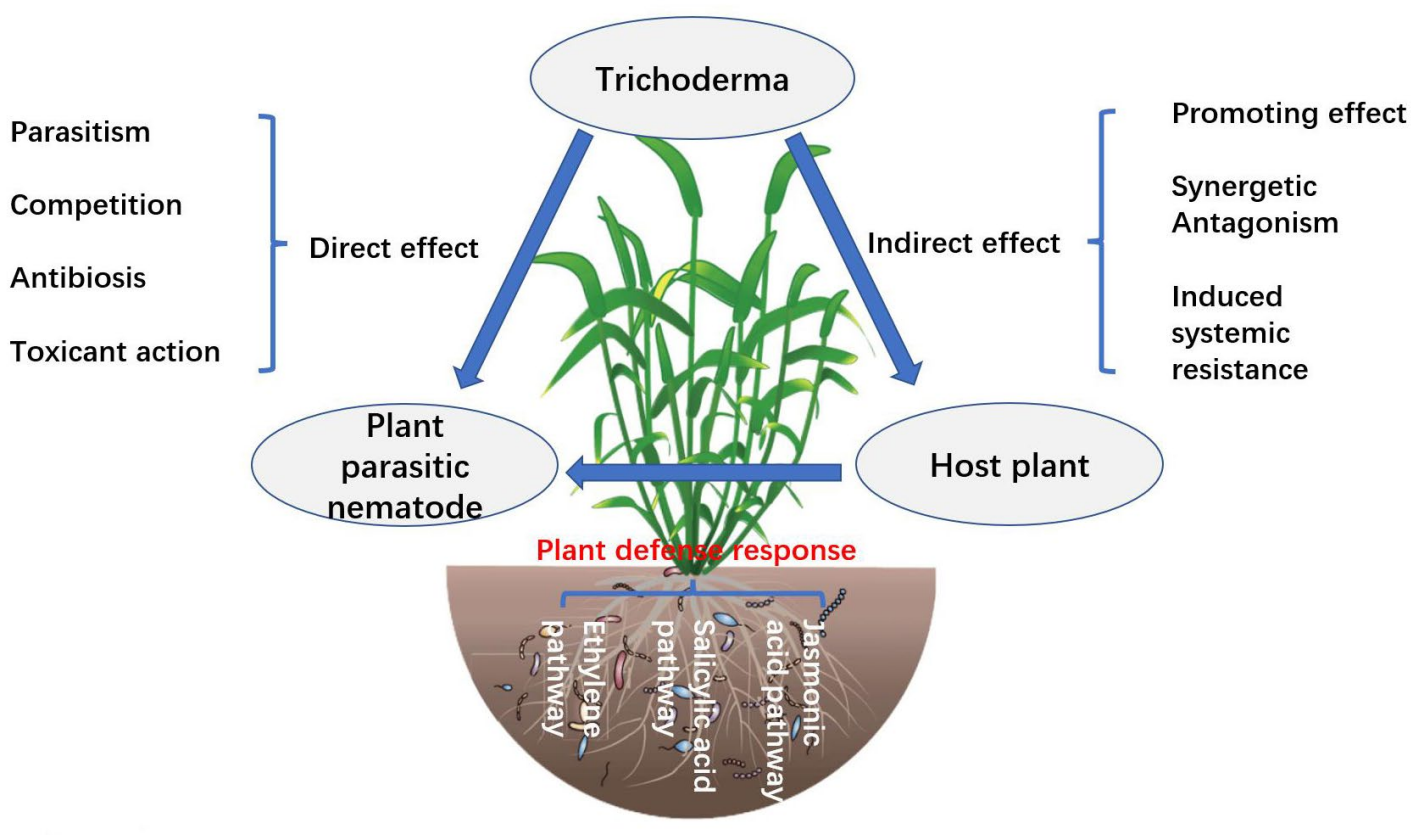
Schematic diagram of the mechanism of action of *Trichoderma* in plant nematode disease control (Sokhandani et al., 2016; Forghani and Hajihassani, 2020; Khan et al., 2020).

*Trichoderma* mycoparasitism is an important mechanism for controlling nematodes and includes identification, contact, entanglement, penetration, and parasitism (Li et al., 2020; Figure 2). *Trichoderma* mycelium penetrates the eggshell or cuticle of larvae and adults of nematodes, colonizes, absorbs nutrients from nematodes, and causes nematode death (Marraschi et al., 2019). *Trichoderma* mycoparasitic nematode processes involve the production and co-secretion of various degrading enzymes (Moo-Koh et al., 2022). The induction of *Trichoderma* activities of β-1, 3-glucanase, chitinase, and protease are increased, which can enhance the immunity of plants to nematodes (Poveda et al., 2020). The hyperparasitic process is mainly regulated by heterotrimeric G protein, cAMP, and MAPK motif signals, and secretes extracellular chitinase, glucanase, xylanase, cellulase, and protease, among which chitinase and protease are particularly important and can degrade the cyst, egg, larva, and adult body wall of nematodes (Li et al., 2020). *Trichoderma* secretes glucosidases NAG1 and NAG2, which act on the extracellular and self-cell walls, respectively, and their main function is to degrade chitin so that they can protect their cell walls from degradation during the process of hyperparasitism (Poveda et al., 2020).

### Antibiosis effect of *Trichoderma*

*Trichoderma* can inhibit the growth and reproduction of nematodes by secreting antagonistic substances (Moo-Koh et al., 2022). *Trichoderma* produces a variety of secondary metabolites, including trichomycin, gliotoxin, viridin, antibacterial peptide, β-1, 3-glucanase, chitinase, polypeptides, polyketones, butyrolactones, sesquiterpene heptadecarboxylic acid, terpenes, and some volatile substances (hydrocarbons, alcohols, furans, aldehydes, alkanes, olefins, esters, aromatic compounds, heterocyclic compounds, and various terpenoids) (Kappel et al., 2020). Contina et al. (2017) reported for the first time that *T. harzianum* ThzID1-M3 labeled with GFP significantly inhibited the reproduction of potato cyst nematodes, with a cyst decline rate of 60%. The inhibition rates of the fermentation broth of *T. hamatum* HZ-9 and *T. virens* HZ-L9 on the hatching of soybean cyst nematode eggs were 80.6 and 79.4%, respectively (Contina et al., 2017). The secondary metabolites produced by the same *Trichoderma* species in different media have different effects on nematode resistance (Forghani and Hajihassani, 2020). The inhibition rate of *T. viride* secondary metabolites on wheat medium and solid medium on egg hatching of southern root knot nematode was 71.6 and 67.3%, respectively (Baazeem et al., 2021). Baazeem et al. (2021) detected and analyzed the *T. hamatum* FB10 secondary metabolite nematicidal active ingredient; thirteen kinds of chemical substances were obtained, including 6-amyl-α-pyranone. The inhibition rate of egg hatching of *Meloidogyne incognita* was 78.26%.

### Toxicity effect of *Trichoderma*

*Trichoderma* produces toxic secondary metabolites that directly come into contact with nematodes, which is an important direct biological control mechanism (Khan et al., 2020). It has been found that the toxic secondary metabolites produced by *Trichoderma* are divided into two categories, one is small molecules and volatile substances, including aromatic compounds, polyketides, butenolactones, and terpenoids, etc.; the other is macromolecular metabolites, including peptides, enzymes, etc. (Khan et al., 2020). It has been reported that the main nematicidal substances isolated from *Trichoderma* are trichodermin, acetic acid, gliotoxin, and peptide cyclosporin A (Fan et al., 2020). Meanwhile, Moo-Koh et al. (2022) used GC–MS to detect and analyze the nematicidal active components of *Trichoderma* TUV-13 strain and obtained more than 40 chemical components, among which of which the main alkanes are the most, in addition to organic acids, esters, ketones, steroids, and other organic compounds. Li et al. (2020) summarized and analyzed the secondary metabolites and activities of 20 species of *Trichoderma*, including *T. aureovirid*e, *T. arundinaceum*, *T. brevicompactum*, *T. citrinoviride*, *T. gamsii*, *T. polysporum*, *T. saturnisporum*, *T. spirale*, *T. cremeum*, *T. pesudokoningii*, and *T. lignorum*. There were 390 non-volatile secondary metabolites, among which wickerol A, harziandione, trichodermin, and cyclonerodiol exhibited nematicidal activity (Khan et al., 2020). The *T. virens* B3 fermentation broth has strong toxic activity against cereal cyst nematodes, and the killing rate is as high as 86.2% (Forghani and Hajihassani, 2020). The fermentation broth can maintain good stability for a long time. The fermentation broth of *T. citrinoviride，T. harzianum，T. acroviride*, and *T. koningiopsis* had a strong toxic effect on the southern root-knot nematode J2, with a mortality rate of more than 85% (Du et al., 2020).

### Induced resistance effect of *Trichoderma*

Induced resistance is the response of plants to stress, which is stimulated by external factors. *Trichoderma*-colonized plant roots cause physiological and metabolic changes and produce a variety of secondary metabolites that act as elicitors (Al-Hazmi and TariqJaveed, 2016). At present, there are more than 20 elicitors produced by *Trichoderma* that induce plant resistance, including antitoxins, polypeptides, lipopeptides, cellulases, hydrophobic proteins, non-toxic gene proteins, terpenoids, phenol derivatives, glycosidic ligands, and flavonoids (Pocurull et al., 2020). These secondary metabolites induce plant defense responses and promote plant growth. The interaction between *Trichoderma* and plants increases the synthesis of defense-related enzymes and substances. *T. hamatum* can induce the activities of phenylalanine ammonia lyase (PAL), polyphenol oxidase (PPO), and peroxidase (POD), which are related to tobacco defense reaction, to increase significantly (Al-Hazmi and TariqJaveed, 2016). In tomatoes treated with *T. harzianum*, the control effect against *M. incognita* was 61.88%. Further studies have shown that the levels of reactive oxygen species (ROS), superoxide (O^2−^), hydrogen peroxide (H_2_O_2_), and malondialdehyde (MDA) in tomatoes were significantly increased, and the defense-related genes PAL, C4H, 4CL, CAD, LPO, CCOMT, Tpx1, and G6PDH were upregulated, thus inducing the defense response of tomatoes to *M. incognita* (Pocurull et al., 2020). Plant-induced resistance mainly involves signal transduction pathways such as those of salicylic acid (SA), jasmonic acid (JA), and ethylene (ET). In the interaction between *Trichoderma* and Arabidopsis, tomato, and cucumber, JA, SA, and ET contents increased to varying degrees, indirectly improving plant resistance (Hinterdobler et al., 2021). This process is also related to activating chitinase and glucanase activities and inhibiting the plant antioxidant enzyme system. The expression of ETR1 and LOX1 genes of jasmonic acid and the ethylene signal pathway increased significantly in *T. asperellum* DQ-1 irrigated tomato, which enhanced tomato resistance (Agbessenou et al., 2022). Some volatile secondary metabolites of *Trichoderma* are important elicitors that induce plant resistance (Al-Hazmi and TariqJaveed, 2016). The volatile substances produced by *T. harzianum* and *T. asperellum* act as elicitors to stimulate the up-regulated expression of Arabidopsis-induced resistance-related transcription factor MYB72, which triggers a JA-regulated defense response (Agbessenou et al., 2022). At present, the interaction mechanisms and signal transduction pathways between *Trichoderma* volatile secondary metabolites and plants have not been thoroughly studied. The active substances produced by *Trichoderma* are recognized by plants, thus activating the signal transduction pathway and inducing the production of plant system resistance. The microbial determinants recognized by microorganisms are called microbe-associated molecular patterns (MAMPs) (Baazeem et al., 2021). After *Trichoderma* infects plant roots, it releases a variety of MAMPs to activate immune response (MTI), thus inducing plant systemic resistance (ISR) (Li X. et al., 2022). Abdelkhalek et al. (2022) showed that *T. hamatum* strain Th23 promotes tomato growth and induces systemic resistance against tobacco mosaic virus.

## Application and mechanism of action of *Trichoderma* in promoting crop growth and repairing environment

### Application of *Trichoderma* in promoting plant growth and repairing environment

*Trichoderma* can produce plant growth stimulators, such as indoleacetic acid (IAA) and harzianolide, to promote the development and growth of plant roots by secreting phytase and ferritin to promote the absorption of P and Fe by plants; decomposes soil organic matter; increases the supply of soil nutrients; improves crop photosynthetic efficiency; improves plant height, stem diameter, and other agronomic traits; and increases production (Lombardi et al., 2020a). Many studies have shown that most *Trichoderma* spp. can produce bioactive substances and have antagonistic effects on plant-pathogenic fungi and plant-pathogenic nematodes (Şesan et al., 2020; Abdelkhalek et al., 2022; Organo et al., 2022; Rao et al., 2022). Bioactive substances, including secondary metabolites and cell wall-degrading enzymes, can effectively improve the resistance of crops, reduce plant diseases, and promote plant growth (Domínguez et al., 2016; Viriyasuthee et al., 2019; Jaiswal et al., 2020; Tseng et al., 2020).

*Trichoderma* can improve soil nutrient availability and utilization efficiency. The aboveground biomass of cucumber seedlings inoculated with *Trichoderma* MF-2 increased by 39.07%, with a significant growth-promoting effect, and an increased number of beneficial microorganisms in the soil (Singh et al., 2019; Ye et al., 2020). Different *Trichoderma* strains had different degrees of antagonism to *F. oxysporum*, and the combination of *Trichoderma* wettable powder treatment significantly increased banana yield (Samuelian, 2016; Bubici et al., 2019; Damodaran et al., 2020). Li et al. (2020) found that the biocontrol agent *Trichoderma* GYXM-1p1 strain had a strong growth-promoting effect through pot cultivation. After treatment with this strain, the root length, plant height, root fresh weight, dry weight, total fresh weight, and total dry weight of cabbage plants were significantly improved compared to the water control (*p* < 0.05), and the total fresh weight and total dry weight of cabbage plants were increased by 417. 60% and 762. 69%, respectively, compared with water control. Ruan et al. (Intana et al., 2021; Nuangmek et al., 2021) found that the application of nitrogen fertilizer with *Trichoderma* promoted the quality of muskmelon. After the application of *Trichoderma*, the soluble sugar content of muskmelon fruit increases significantly, improving the quality of muskmelon. The application of *Trichoderma* can increase the SPAD value of chlorophyll in peanut leaves, improve the main agronomic traits of peanuts, significantly increase the activity of protective enzymes in peanut roots, stems, and leaves, and reduce the content of MDA (Kovács et al., 2021; Al-Askar et al., 2022). When 1.5 kg/666.7 m^−2^ was applied, the number of pods per plant, pod weight, seed kernel weight, 100 fruit quality, 100 fruit kernel quality, and yield per plant of peanut increased by 24.63, 20.22, 14.10, 4.86, 7.63, and 12.85%, respectively, compared with the control (Al-Askar et al., 2022).

### Mechanism of *Trichoderma* in promoting plant growth and repairing environment

**Figure 3:**
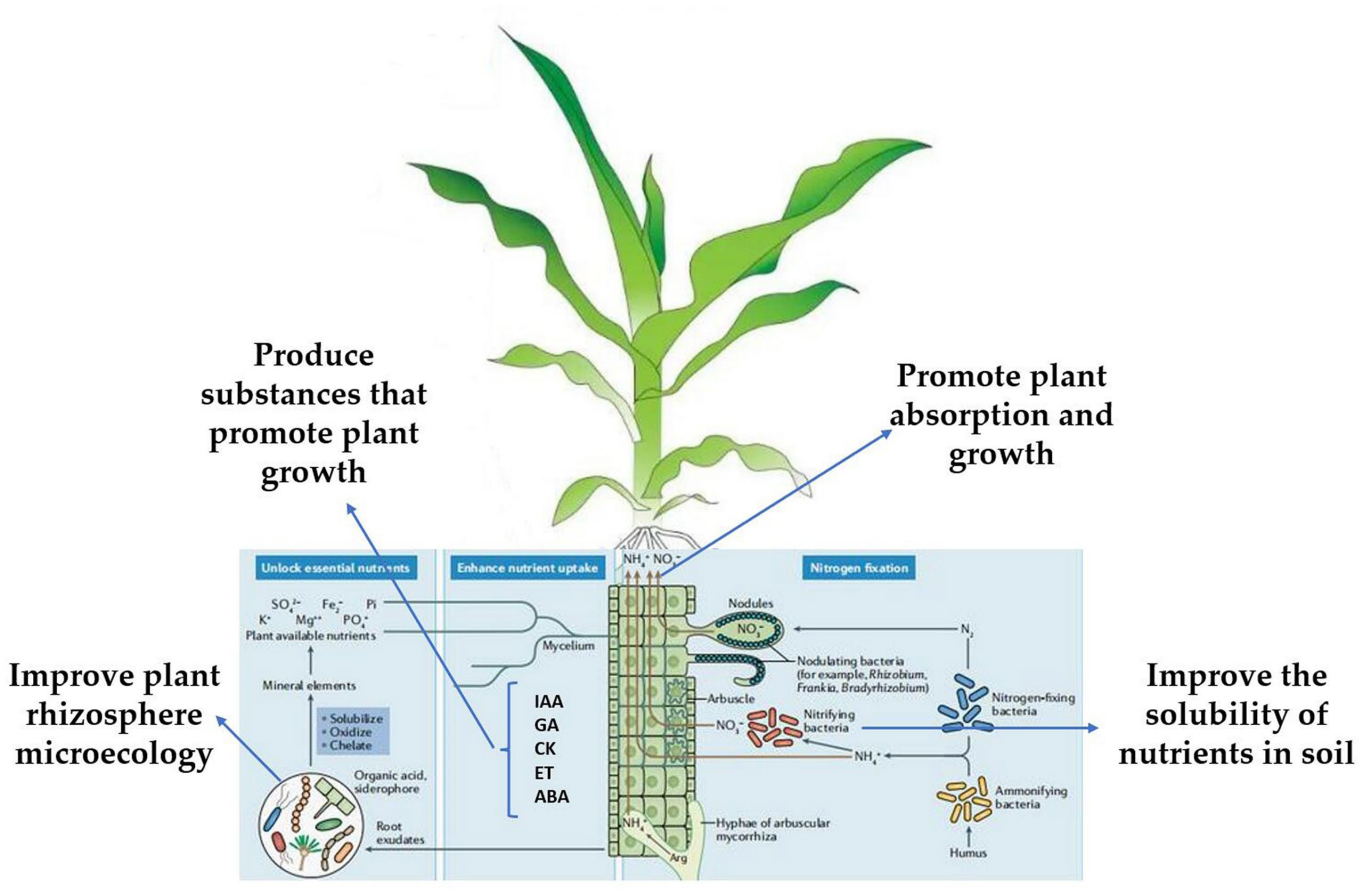
Schematic diagram of the mechanism of action of *Trichoderma* in promoting crop growth and repairing the environment (Jaiswal et al., 2020; Lombardi et al., 2020a; Şesan et al., 2020; Karuppiah et al., 2021).

*Trichoderma* can promote plant growth, produce substances that can promote plant growth, improve the solubility of nutrients in the soil, and improve plant rhizosphere microecology, thereby promoting plant absorption and growth (Karuppiah et al., 2019a; Kakabouki et al., 2021; Marra et al., 2021; Figure 3). *Trichoderma* plant interactions can not only induce resistance but also improve the resistance of plants to abiotic stress factors (salt, high temperature, UV). Treatment of cucumber seeds with *T. asperellum* T203 improved the plant’s salt tolerance, and the activities of Mn/Cu SOD and catalase (CAT), and significantly reduced ascorbic acid in the plant (Illescas et al., 2022). *Trichoderma* can significantly enhance the Na^+^ efflux from the root system of *Lycium barbarum* and its transport to the upper ground, ensure K^+^ absorption and maintain the ion balance in the plant, thus reducing the damage of PSII caused by ion toxicity and oxidative stress, protecting photosynthetic pigments, maintaining the photosynthetic performance of *L. barbarum* under salt stress, and reducing biomass loss (Brotman et al., 2013).

The synthesis of plant growth hormones, such as IAA, ABA, ET, GA, and CK is the main mechanism of *Trichoderma* (Karuppiah et al., 2019b; Wang et al., 2021; Degani et al., 2021b; Agbessenou et al., 2022). *T. asperellum* induced cucumber to produce IAA, GA, and ABA to promote growth (Liu H. et al., 2022). The height, stem diameter, soluble sugar content, and absorption rate of available nitrogen of tomato seedlings treated with *T. asperellum* were significantly increased, and the expression of tomato hormone signal transduction-related genes JAR1, MYC2, NPR1, PR1, and GH3 was significantly increased (Rawal et al., 2022). Another study showed that *T. asperellum* can upregulate the expression of xylanase genes in poplar and has a significant growth-promoting effect (Karuppiah et al., 2019b). *T. harzianum* regulates tricarboxylic acid cycle (TAC) and hexose monophosphate pathway (HMP) to promote tomato growth by enhancing succinate dehydrogenase and glucose-6-phosphate dehydrogenase activities (Manganiello et al., 2018). *Trichoderma* produces acidic substances that can dissolve insoluble trace elements in soil and provide more nutrition to plants (Samuelian, 2016). *T. asperellum* can transform insoluble phosphate in the soil into effective phosphorus and promote the absorption and utilization of cucumbers (Karuppiah et al., 2021; Figure 3). *T. koningiopsis* can produce organic acids that dissolve insoluble tricalcium phosphate under high alkaline stress and can also produce alkaline phosphatase under drought stress to solubilize phosphorus and improve the utilization of nutrients by plants (You et al., 2022). Many experiments have verified that *Trichoderma* promotes plant growth (Mayo et al., 2015; Bononi et al., 2020; Lombardi et al., 2020b; Swain et al., 2021; Velasco et al., 2021; Bridžiuvienė et al., 2022; Joo and Hussein, 2022; Li X. et al., 2022); under complex field production conditions, the mechanism by which *Trichoderma* promotes plant growth requires more systematic research.

## Conclusion and future perspectives

At present, chemical control is the main method used for plant disease control and is achieved by spraying pesticides and fungicides. Although the effect of chemical control is good and helpful in increasing agricultural production, the unscientific use of chemical pesticides has caused serious pollution to the environment and enhanced pathogens’ resistance to and chemical pesticides. Several experiments have proven that *Trichoderma* has good biological control effects and can reduce the use of chemical pesticides. However, there are still few biocontrol agents against *Trichoderma* on the market, and more effective and suitable strains need to be found to join the biocontrol team (Nieto-Jacobo et al., 2017; Fiorentino et al., 2018; López et al., 2019; Nawrocka et al., 2019; Poveda et al., 2019; Cabral-Miramontes et al., 2022). Although *Trichoderma* has great prospects for agricultural applications, there are still some problems in the development and utilization of *Trichoderma* (Rubio et al., 2014; Zhang et al., 2018; Phoka et al., 2020; Santos et al., 2020; Wang H. et al., 2022). Because the spore preparation of *Trichoderma* is generally a living fungal preparation, which is often affected by various natural factors such as humidity, temperature, soil acidity, alkalinity, and the soil microbial community when it is applied in the field, the field test performance is unstable, and the biological control effect is weakened. In addition, the shelf life of biological control agents is relatively short, and some microorganisms must be stored at low temperatures to ensure the concentration of live microorganisms when they are applied.

There are still several problems to be solved in the application of *Trichoderma* in the biological control of plant diseases (Caruso et al., 2020; He et al., 2020; Boamah et al., 2021; González-López et al., 2021). The first is to explore and produce efficient strains, which can be screened through genetic engineering technology to produce *Trichoderma* biocontrol-engineered strains that are resistant to chemical pesticides and low temperatures. At the same time, it is necessary to develop effective *Trichoderma* agents suitable for use with various application methods to enhance the control effect and improve the processing technology of *Trichoderma* agents to extend the shelf life of biological control agents. Second, exploring the combined effects of *Trichoderma* spp. and other microorganisms is necessary. The development of Pesticides with volatile and non-volatile secondary metabolites secreted by *Trichoderma* as the main active ingredient will be the focus of future research and the development of new Pesticides. To improve the quality of *Trichoderma* biopesticides, in addition to monitoring traditional indicators such as pH, dissolved oxygen, and temperature in the fermentation process, it is also necessary to monitor its correlation with the yield of antagonistic substances at the level of the cell metabolic flow, genome, proteome, and metabolome (Mulatu et al., 2021). However, it is necessary to establish more scientific quality standards for *Trichoderma* products *in vivo*, such as increasing the content of antagonistic substances or activity indicators (Niu et al., 2020). The development of new dosage forms, such as cell microcapsules, water-in-oil emulsions, and other protective dosage forms, should be strengthened, and the molecular mechanism of chlamydospore production should be further studied (Table 1).

**Table 1:** The main biological function of common species of *Trichoderma.*

Name of species	Major function	Reference
*T. harzianum*	Induced systemic resistance to fungi disease and increased plant productivity; Nematode resistance; Improved plant growth and root architecture.	Saravanakumar et al. (2016), Poveda et al. (2019), Coppola et al. (2019)
*T. asperellum*	Antifungal activities; Plant growth promotion; Stress resistance; Enrich soil fertility	Wang H. et al. (2022), AL-Askar et al. (2021), Degani et al. (2021b)
*T. asperelloides*	Antifungal activities; Plant growth promotion; Stress resistance	Ruangwong et al. (2021a), Phoka et al. (2020), Rawal et al. (2022)
*T. atroviride*	Fungistatic activity, plant growth promotion; Antifungal and antibacterial activities; Plant growth promotion and nutrient assimilation; Induced systemic defense responses; Stress resistance	Coppola et al. (2019), Zhang C. et al. (2022), González-López et al. (2021), Leal et al. (2021), Nawrocka et al. (2019)
*T. hamatum*	Nematode resistance; Increased plant productivity; Antibacterial and antifungal activities; Plant growth promoting	Li X. et al. (2022), Velasco et al. (2021), Baazeem et al. (2021)
*T. virens*	Antifungal activities; Plant growth promotion	Jogaiah et al. (2018), Halifu et al. (2020)
*T. viride*	Antifungal activities; Enhanced root development; Nematode resistance; Stress resistance	Al-Hazmi and TariqJaveed, (2016), Naglot et al. (2015), He et al. (2020)
*T. longibrachiatum*	Antifungal activities; Improve salt resistance; Nematode resistance; Plant growth promotion; Induced systemic defense responses	Ngo et al. (2021), Boamah et al. (2021), AL-Askar et al. (2022), Degani et al. (2021b), Yuan et al. (2019)
*T. ghanense*	Plant growth promotion; Enrich soil fertility	Bridžiuvienė et al., 2022
*T. tomentosum*	Plant growth promotion; Enrich soil fertility	Bridžiuvienė et al. (2022)
*T. volatiles*	Induced systemic resistance	Pescador et al. (2022)
*T. velutinum*	Plant growth promotion	Mayo-Prieto et al. (2019)
*T. phayaoense*	Antifungal activities; improve plant growth and root architecture	Nuangmek et al. (2021)
*T. koningiopsis*	Antifungal activities; Plant growth promotion	Ruangwong et al. (2021b), You et al. (2022)
*T. citrinoviride*	Antifungal activities; Nematode resistance	Park et al. (2019), Fan et al. (2020)
*T. reesei*	Antifungal activities	Hinterdobler et al. (2021)
*T. gamsii*	Antifungal activities; Affected herbivore feeding behavior	Alukumbura et al. (2022), Zhou et al. (2018), Di Marco et al. (2022)
*T. aggressivum*	Fungal diseases biological control	Sánchez-Montesinos et al. (2021)
*T. atrobrunneum*	Nematode resistance	Hernández et al. (2018)
*T. afroharzianum*	Plant growth promotion	Kappel et al. (2022)
*T. bissettii*	Antifungal activities	Chung et al. (2022)
*T. parareesei*	Improve plant quality	Rubio et al. (2014)
*T. lignorum*	Nematode resistance	Daza et al. (2019)
*T. taxi*	Antifungal activities	Chen et al. (2021)
*T. strigosellum*	Nematode resistance; Plant growth promotion	Batista et al. (2021)
*T. hebeiensis*	Antifungal activities; Plant growth promotion	Swain et al. (2021)
*T. erinaceum*	Antifungal activities; Plant growth promotion	Swain et al. (2021)

With further study of transgenic *Trichoderma*, a prospective study on the biological and environmental safety of transgenic *Trichoderma* should be conducted (Li et al., 2021). At present, the balance regulation of *Trichoderma* colonizing host and plant immune response, long-distance and trans-growth period transduction mechanism of systematically induced plant disease resistance and its defense signals, identification of *Trichoderma* elicitors to recognize plant targets or receptors, and mechanism of *Trichoderma*-induced plant endophytic microbiome to synergistically stimulate plant immune response has become an international research topic of interest. Studies on miRNA regulation of *Trichoderma* colonization host process and plant immune response, and the regulation mechanism of cross-border miRNA transduction between *Trichoderma*, plants, and pathogenic microorganisms are emerging. The combination design or co-culture technology of *Trichoderma* and other microorganisms has become key for tapping new metabolites with specific functions of microorganisms, broadening the target spectrum of microbial metabolites, and developing new biopesticides and biostimulants based on metabolites (Wang Y. et al., 2022). It is expected to become a new direction for the development of macromolecular biopesticides by molecular construction or modification of the *Trichoderma* multi-stimulator fusion protein and the development of new plant immune-activating protein pesticides. At present, it is urgent to reveal the synergistic interactions among *Trichoderma*, plants, and pathogenic microorganisms in induced disease resistance on a cross-genome scale, develop *Trichoderma* and other microbial symbiotic agents that can cure both diseases and pests, and develop new biostimulator products based on *Trichoderma* metabolites.

Compared with single-life biocontrol fungi, compound biocontrol fungi can better avoid the problems of weak adaptability to the environment, narrow range of disease resistance, and insufficient control effect. At present, there are many preparations containing different kinds of *Trichoderma*, which are being used in sustainable agricultural crops, but the application of these preparations is still expensive, and not available to all farmers. The application of compatible or affinity multiple microorganisms for compounding has become a trend in the development of biocontrol agents. *Trichoderma* can form alliances with a variety of microorganisms such as bacteria and fungi to directly or indirectly improve the ability of plants to prevent and control diseases. The following aspects may be the main research focus of *Trichoderma* as a biocontrol fungus in the future: the molecular mechanism of the specific interaction between *Trichoderma* and plants; Molecular basis of plant immunity induced by *Trichoderma*; Improvement of *Trichoderma* fermentation process; Establishing diversified application technology models of *Trichoderma*. The commercial application of biocontrol *Trichoderma* depends largely on the stress resistance (such as high temperature, drying, ultraviolet radiation, etc.) and storage resistance (more than 1 year at normal temperature) of the *Trichoderma* agent. At present, there are two main technologies. One is to reduce the acidity and regulate the utilization of oxygen to induce *Trichoderma* to produce stress-resistant chlamydospores, and the other is to add some chemical additives (such as copper) to the inoculum. How the effectors produced by *Trichoderma* interact with plant cell receptors has become the key to revealing the mechanism of *Trichoderma* inducing plant immunity. With the deepening of the research on transgenic *Trichoderma*, prospective research on the biological and environmental safety of transgenic *Trichoderma* should be carried out.

## Author contributions

MZ and XY: software. KZ and MZ: data curation. XY: writing – original draft preparation. JR, JC, and KZ: writing, review, and editing. All authors read and agreed to the published version of the manuscript.

## Funding

This research was supported by the National Science Foundation of China (32161143005 and 32160669) and the Guizhou Science and Technology Support Program ([2020]1Y125).

## Conflict of interest

The authors declare that the research was conducted in the absence of any commercial or financial relationships that could be construed as a potential conflict of interest.

## Publisher’s note

All claims expressed in this article are solely those of the authors and do not necessarily represent those of their affiliated organizations, or those of the publisher, the editors and the reviewers. Any product that may be evaluated in this article, or claim that may be made by its manufacturer, is not guaranteed or endorsed by the publisher.

## References

[ref1] Abbas A. Mubeen M. Zheng H. Sohail M. A. Shakeel Q. Solanki M. K.. (2022). *Trichoderma* spp. genes involved in the biocontrol activity against *Rhizoctonia solani*. Front. Microbiol. 13:884469. doi: 10.3389/fmicb.2022.884469, PMID: 35694310 PMC9174946

[ref2] Abdelkhalek A. Al-Askar A. A. Arishi A. A. Behiry S. I. (2022). *Trichoderma hamatum* strain Th23 promotes tomato growth and induces systemic resistance against tobacco mosaic virus. J. Fungi 8:228. doi: 10.3390/jof8030228, PMID: 35330230 PMC8951347

[ref3] Agbessenou A. Akutse K. S. Yusuf A. A. Khamis F. M. (2022). The endophyte *Trichoderma asperellum* M2RT4 induces the systemic release of methyl salicylate and (Z)-jasmone in tomato plant affecting host location and herbivory of *Tuta absoluta*. Front. Plant Sci. 13:860309. doi: 10.3389/fpls.2022.860309, PMID: 35449888 PMC9016226

[ref4] Al-Askar A. A. Rashad E. M. Moussa Z. Ghoneem K. M. Mostafa A. A. Al-Otibi F. O.. (2022). A novel endophytic *Trichoderma longibrachiatum* WKA55 with biologically active metabolites for promoting germination and reducing mycotoxinogenic fungi of peanut. Front. Microbiol. 13:772417. doi: 10.3389/fmicb.2022.772417, PMID: 35401430 PMC8993229

[ref5] Al-Askar A. A. Saber W. Ghoneem K. M. Hafez E. E. Ibrahim A. A. (2021). Crude citric acid of *Trichoderma asperellum*: tomato growth promotor and suppressor of *Fusarium oxysporum* f. sp. *lycopersici*. Plants 10:222. doi: 10.3390/plants10020222, PMID: 33498925 PMC7912305

[ref6] Alfiky A. Weisskopf L. (2021). Deciphering *Trichoderma*-plant-pathogen interactions for better development of biocontrol applications. J. Fungi 7:61. doi: 10.3390/jof7010061, PMID: 33477406 PMC7830842

[ref7] Al-Hazmi A. S. TariqJaveed M. (2016). Effects of different inoculum densities of *Trichoderma harzianum* and *Trichoderma viride* against *Meloidogyne javanica* on tomato. Saudi J. Biol. Sci. 23, 288–292. doi: 10.1016/j.sjbs.2015.04.007, PMID: 26981012 PMC4778584

[ref8] Alonso-Ramírez A. Poveda J. Martín I. Hermosa R. Monte E. Nicolás C. (2014). Salicylic acid prevents *Trichoderma harzianum* from entering the vascular system of roots. Mol. Plant Pathol. 15, cc doi: 10.1111/mpp.12141, PMID: 24684632 PMC6638820

[ref9] Al-Surhanee A. A. (2022). Protective role of antifusarial eco-friendly agents (*Trichoderma* and salicylic acid) to improve resistance performance of tomato plants. Saudi J. Biol. Sci. 29, 2933–2941. doi: 10.1016/j.sjbs.2022.01.020, PMID: 35531256 PMC9073055

[ref10] Alukumbura A. S. Bigi A. Sarrocco S. Fernando W. Vannacci G. Mazzoncini M.. (2022). Minimal impacts on the wheat microbiome when *Trichoderma gamsii* T6085 is applied as a biocontrol agent to manage fusarium head blight disease. Front. Microbiol. 13:972016. doi: 10.3389/fmicb.2022.972016, PMID: 36212885 PMC9539683

[ref11] Álvarez-García S. Mayo-Prieto S. Gutiérrez S. Casquero P. A. (2020). Self-inhibitory activity of *Trichoderma* soluble metabolites and their antifungal effects on *Fusarium oxysporum*. J. Fungi 6:176. doi: 10.3390/jof6030176, PMID: 32957718 PMC7559637

[ref12] Andrade-Hoyos P. Silva-Rojas H. V. Romero-Arenas O. (2020). Endophytic *Trichoderma* species isolated from *Persea americana* and *Cinnamomum verum* roots reduce symptoms caused by *Phytophthora cinnamomi* in avocado. Plan. Theory 9:1220. doi: 10.3390/plants9091220, PMID: 32957543 PMC7569818

[ref001] Aswani R. Roshmi T. Radhakrishnan E. K. (2022). Induction of plant defense response by endophytic microorganisms. In Biocontrol Mechanisms of Endophytic Microorganisms. Academic Press. 89–115., PMID:

[ref13] Baazeem A. Almanea A. Manikandan P. Alorabi M. Vijayaraghavan P. Abdel-Hadi A. (2021). In vitro antibacterial, antifungal, nematocidal and growth promoting activities of *Trichoderma hamatum* FB10 and its secondary metabolites. J. Fungi 7:331. doi: 10.3390/jof7050331, PMID: 33923354 PMC8145006

[ref14] Bardin M. Ajouz S. Comby M. Lopez-Ferber M. Graillot B. Siegwart M.. (2015). Is the efficacy of biological control against plant diseases likely to be more durable than that of chemical pesticides? Front. Plant Sci. 6:566. doi: 10.3389/fpls.2015.00566, PMID: 26284088 PMC4515547

[ref15] Basińska-Barczak A. Błaszczyk L. Szentner K. (2020). Plant cell wall changes in common wheat roots as a result of their interaction with beneficial fungi of *Trichoderma*. Cells 9:2319. doi: 10.3390/cells9102319, PMID: 33086614 PMC7603241

[ref16] Bazghaleh N. Prashar P. Woo S. Vandenberg A. (2020). Effects of lentil genotype on the colonization of beneficial *Trichoderma* species and biocontrol of aphanomyces root rot. Microorganisms 8:1290. doi: 10.3390/microorganisms8091290, PMID: 32846963 PMC7564536

[ref007] Batista K. O. M. Silva D. V. Nascimento V. L. de Souza D. J. (2021). Effects of *Trichoderma strigosellum* in eucalyptus urophylla development and leaf-cutting ant behavior. J Fungi 8:15., PMID: 35049955 10.3390/jof8010015 PMC8778871

[ref17] Boamah S. Zhang S. Xu B. Li T. Calderón-Urrea A. (2021). *Trichoderma longibrachiatum* (TG1) enhances wheat seedlings tolerance to salt stress and resistance to *Fusarium pseudograminearum*. Front. Plant Sci. 12:741231. doi: 10.3389/fpls.2021.741231, PMID: 34868125 PMC8635049

[ref18] Bononi L. Chiaramonte J. B. Pansa C. C. Moitinho M. A. Melo I. S. (2020). Phosphorus-solubilizing *Trichoderma* spp. from Amazon soils improves soybean plant growth. Sci. Rep. 10:2858. doi: 10.1038/s41598-020-59793-8, PMID: 32071331 PMC7028723

[ref19] Bridžiuvienė D. Raudonienė V. Švedienė J. Paškevičius A. Baužienė I. Vaitonis G.. (2022). Impact of soil chemical properties on the growth promotion ability of *Trichoderma ghanense*, *T. tomentosum* and their complex on Rye in different land-use systems. J. Fungi 8:85. doi: 10.3390/jof8010085, PMID: 35050025 PMC8777797

[ref20] Brotman Y. Landau U. Cuadros-Inostroza Á. Tohge T. Fernie A. R. Chet I.. (2013). *Trichoderma*-plant root colonization: escaping early plant defense responses and activation of the antioxidant machinery for saline stress tolerance. PLoS Pathog. 9:e1003221. doi: 10.1371/journal.ppat.1003221, PMID: 23516362 PMC3597500

[ref21] Bubici G. Kaushal M. Prigigallo M. I. Gómez-Lama Cabanás C. Mercado-Blanco J. (2019). Biological control agents against *Fusarium* wilt of Banana. Front. Microbiol. 10:616. doi: 10.3389/fmicb.2019.00616, PMID: 31024469 PMC6459961

[ref22] Cabral-Miramontes J. P. Olmedo-Monfil V. Lara-Banda M. Zúñiga-Romo E. R. Aréchiga- Carvajal E. T. (2022). Promotion of plant growth in arid zones by selected *Trichoderma* spp. strains with adaptation plasticity to alkaline pH. Biology 11:1206. doi: 10.3390/biology11081206, PMID: 36009833 PMC9405189

[ref23] Cai X. Zhao H. Liang C. Li M. Liu R. (2021). Effects and mechanisms of symbiotic microbial combination agents to control tomato *Fusarium* crown and root rot disease. Front. Microbiol. 12:629793. doi: 10.3389/fmicb.2021.629793, PMID: 34220730 PMC8245789

[ref24] Carro-Huerga G. Compant S. Gorfer M. Cardoza R. E. Schmoll M. Gutiérrez S.. (2020). Colonization of *Vitis vinifera* L. by the endophyte *Trichoderma* sp. strain T154: biocontrol activity against *Phaeoacremonium minimum*. Front. Plant Sci. 11:1170. doi: 10.3389/fpls.2020.01170, PMID: 32849725 PMC7417607

[ref25] Caruso G. El-Nakhel C. Rouphael Y. Comite E. Lombardi N. Cuciniello A.. (2020). *Diplotaxis tenuifolia* (L.) DC. Yield and quality as influenced by cropping season, protein hydrolysates, and *Trichoderma* applications. Plan. Theory 9:697. doi: 10.3390/plants9060697, PMID: 32486184 PMC7356635

[ref26] Chen J. Zhou L. Din I. U. Arafat Y. Li Q. Wang J.. (2021). Antagonistic activity of *Trichoderma* spp. against *Fusarium oxysporum* in rhizosphere of radix pseudostellariae triggers the expression of host defense genes and improves its growth under long-term monoculture system. Front. Microbiol. 12:579920. doi: 10.3389/fmicb.2021.781826, PMID: 33790872 PMC8005620

[ref27] Chung D. Kwon Y. M. Lim J. Y. Bae S. S. Choi G. Lee D. S. (2022). Characterization of chitinolytic and antifungal activities in marine-derived *Trichoderma bissettii* strains. Mycobiology 50, 244–253. doi: 10.1080/12298093.2022.2105509, PMID: 36158047 PMC9467547

[ref28] Contina J. B. Dandurand L. M. Knudsen G. R. (2017). Use of GFP-tagged *Trichoderma harzianum* as a tool to study the biological control of the potato cyst nematode *Globodera pallida*. Appl. Soil Ecol. 115, 31–37. doi: 10.1016/j.apsoil.2017.03.010

[ref003] Coppola. Diretto G. Digilio M. C. Woo S. L. Giuliano G. Molisso D.. (2019). Transcriptome and metabolome reprogramming in tomato plants by *Trichoderma harzianum* strain T22 primes and enhances defense responses against aphids. Front. Physiol. 10:745., PMID: 31293434 10.3389/fphys.2019.00745 PMC6599157

[ref29] Damodaran T. Rajan S. Muthukumar M. Gopal R. Yadav K. Kumar S.. (2020). Biological management of banana Fusarium wilt caused by *Fusarium oxysporum* f. sp. cubense tropical race 4 using antagonistic fungal isolate CSR-T-3 (*Trichoderma reesei*). Front. Microbiol. 11:595845. doi: 10.3389/fmicb.2020.595845, PMID: 33391212 PMC7772460

[ref006] Daza F. F. F. Roman G. R. Rodriguez M. V. Vargas I. A. G. Heano H. C. Cereda M. P.. (2019). Spores of *Beauveria bassiana* and *Trichoderma lignorum* as a bioinsecticide for the control of atta cephalotes. Biol Res. 52:51., PMID: 31530279 10.1186/s40659-019-0259-y PMC6749709

[ref30] De Zotti M. Sella L. Bolzonello A. Gabbatore L. Peggion C. Bortolotto A.. (2020). Targeted amino acid substitutions in a *Trichoderma* Peptaibol confer activity against fungal plant pathogens and protect host tissues from *Botrytis cinerea* infection. Int. J. Mol. Sci. 21:7521. doi: 10.3390/ijms21207521, PMID: 33053906 PMC7589190

[ref31] Debbi A. Boureghda H. Monte E. Hermosa R. (2018). Distribution and genetic variability of *Fusarium oxysporum* associated with tomato diseases in Algeria and a biocontrol strategy with indigenous *Trichoderma* spp. Front. Microbiol. 9:282. doi: 10.3389/fmicb.2018.00282, PMID: 29515557 PMC5826367

[ref32] Debode J. De Tender C. Cremelie P. Lee A. S. Kyndt T. Muylle H.. (2018). *Trichoderma*- inoculated miscanthus straw can replace peat in strawberry cultivation, with beneficial effects on disease control. Front. Plant Sci. 9:213. doi: 10.3389/fpls.2018.00213, PMID: 29515613 PMC5826379

[ref33] Degani O. Dor S. (2021). *Trichoderma* biological control to protect sensitive maize hybrids against late wilt disease in the field. J. Fungi 7:315. doi: 10.3390/jof7040315, PMID: 33919659 PMC8073241

[ref34] Degani O. Khatib S. Becher P. Gordani A. Harris R. (2021a). *Trichoderma asperellum* secreted 6-pentyl-α-pyrone to control *Magnaporthiopsis maydis*, the maize late wilt disease agent. Biology 10:897. doi: 10.3390/biology10090897, PMID: 34571774 PMC8470384

[ref35] Degani O. Rabinovitz O. Becher P. Gordani A. Chen A. (2021b). *Trichoderma longibrachiatum* and *Trichoderma asperellum* confer growth promotion and protection against late wilt disease in the field. J. Fungi 7:444. doi: 10.3390/jof7060444, PMID: 34199413 PMC8229153

[ref36] Di Marco S. Metruccio E. G. Moretti S. Nocentini M. Carella G. Pacetti A.. (2022). Activity of *Trichoderma asperellum* strain ICC 012 and *Trichoderma gamsii* strain ICC 080 toward diseases of esca complex and associated pathogens. Front. Microbiol. 12:813410. doi: 10.3389/fmicb.2021.813410, PMID: 35154039 PMC8831765

[ref37] Domínguez S. Rubio M. B. Cardoza R. E. Gutiérrez S. Nicolás C. Bettiol W.. (2016). Nitrogen metabolism and growth enhancement in tomato plants challenged with *Trichoderma harzianum* expressing the *Aspergillus nidulans* acetamidase *amdS* gene. Front. Microbiol. 7:1182. doi: 10.3389/fmicb.2016.01182, PMID: 27536277 PMC4971021

[ref38] Druzhinina I. S. Chenthamara K. Zhang J. Atanasova L. Yang D. Miao Y.. (2018). Massive lateral transfer of genes encoding plant cell wall-degrading enzymes to the mycoparasitic fungus *Trichoderma* from its plant- associated hosts. PLoS Genet. 14:e1007322. doi: 10.1371/journal.pgen.1007322, PMID: 29630596 PMC5908196

[ref39] Du F. Y. Ju G. L. Xiao L. Zhou Y. M. Wu X. (2020). Sesquiterpenes and cyclodepsipeptides from marine-derived fungus *Trichoderma longibrachiatum* and their antagonistic activities against soil-borne pathogens. Mar. Drugs 18:165. doi: 10.3390/md18030165, PMID: 32188169 PMC7142749

[ref40] Dugassa A. Alemu T. Woldehawariat Y. (2021). In-vitro compatibility assay of indigenous *Trichoderma* and *Pseudomonas* species and their antagonistic activities against black root rot disease (*Fusarium solani*) of faba bean (*Vicia faba* L.). BMC Microbiol. 21:115. doi: 10.1186/s12866-021-02181-7, PMID: 33865331 PMC8052857

[ref41] El-Hasan A. Walker F. Klaiber I. Schöne J. Pfannstiel J. Voegele R. T. (2022). New approaches to manage Asian soybean rust (*Phakopsora pachyrhizi*) using *Trichoderma* spp. or their antifungal secondary metabolites. Meta 12:507. doi: 10.3390/metabo12060507, PMID: 35736440 PMC9227527

[ref42] Fan H. Yao M. Wang H. Zhao D. Zhu X. Wang Y.. (2020). Isolation and effect of *Trichoderma citrinoviride* Snef1910 for the biological control of root-knot nematode, *Meloidogyne incognita*. BMC Microbiol. 20:299. doi: 10.1186/s12866-020-01984-4, PMID: 33008296 PMC7531111

[ref43] Ferreira Filho J. A. Horta M. A. C. Dos Santos C. A. Almeida D. A. Murad N. F. Mendes J. S.. (2020). Integrative genomic analysis of the bioprospection of regulators and accessory enzymes associated with cellulose degradation in a filamentous fungus (*Trichoderma harzianum*). BMC Genomics 21:757. doi: 10.1186/s12864-020-07158-w, PMID: 33138770 PMC7607812

[ref44] Filizola P. Luna M. de Souza A. F. Coelho I. L. Laranjeira D. Campos-Takaki G. M. (2019). Biodiversity and phylogeny of novel *Trichoderma* isolates from mangrove sediments and potential of biocontrol against *Fusarium* strains. Microb. Cell Factories 18:89. doi: 10.1186/s12934-019-1108-y, PMID: 31122261 PMC6532204

[ref45] Fiorentino N. Ventorino V. Woo S. L. Pepe O. De Rosa A. Gioia L.. (2018). *Trichoderma*-based biostimulants modulate rhizosphere microbial populations and improve N uptake efficiency, yield, and nutritional quality of leafy vegetables. Front. Plant Sci. 9:743. doi: 10.3389/fpls.2018.00743, PMID: 29922317 PMC5996573

[ref46] Fontana D. C. de Paula S. Torres A. G. de Souza V. Pascholati S. F. Schmidt D.. (2021). Endophytic fungi: biological control and induced resistance to phytopathogens and abiotic stresses. Pathogens 10:570. doi: 10.3390/pathogens10050570, PMID: 34066672 PMC8151296

[ref47] Forghani F. Hajihassani A. (2020). Recent advances in the development of environmentally benign treatments to control root-knot nematodes. Front. Plant Sci. 11:1125. doi: 10.3389/fpls.2020.01125, PMID: 32793271 PMC7387703

[ref48] Girma A. (2022). In vitro biocontrol evaluation of some selected *Trichoderma* strains against the root pathogen *Fusarium oxysporum* of hot pepper (*Capsicum annum* L.) in Bure Woreda, Ethiopia. Int. J. Microbiol. 2022:1664116. doi: 10.1155/2022/1664116, PMID: 35880205 PMC9308519

[ref49] Gomes E. V. Costa M. de Paula R. G. de Azevedo R. R. da Silva F. L. Noronha E. F.. (2015). The Cerato-Platanin protein Epl-1 from *Trichoderma harzianum* is involved in mycoparasitism, plant resistance induction and self-cell wall protection. Sci. Rep. 5:17998. doi: 10.1038/srep17998, PMID: 26647876 PMC4673615

[ref50] González-López M. Jijón-Moreno S. Dautt-Castro M. Ovando-Vázquez C. Ziv T. Horwitz B. A.. (2021). Secretome analysis of *Arabidopsis*-*Trichoderma atroviride* interaction unveils new roles for the plant glutamate: Glyoxylate aminotransferase GGAT1 in plant growth induced by the fungus and resistance against *Botrytis cinerea*. Int. J. Mol. Sci. 22:6804. doi: 10.3390/ijms22136804, PMID: 34202732 PMC8268252

[ref51] Guo Y. Ghirardo A. Weber B. Schnitzler J. P. Benz J. P. Rosenkranz M. (2019). *Trichoderma* species differ in their volatile profiles and in antagonism toward ectomycorrhiza *Laccaria bicolor*. Front. Microbiol. 10:891. doi: 10.3389/fmicb.2019.00891, PMID: 31105677 PMC6499108

[ref52] Guzmán-Guzmán P. Alemán-Duarte M. I. Delaye L. Herrera-Estrella A. Olmedo-Monfil V. (2017). Identification of effector- like proteins in *Trichoderma* spp. and role of a hydrophobin in the plant-fungus interaction and mycoparasitism. BMC Genet. 18:16. doi: 10.1186/s12863-017-0481-y, PMID: 28201981 PMC5310080

[ref53] Halifu S. Deng X. Song X. Song R. Liang X. (2020). Inhibitory mechanism of *Trichoderma virens* ZT05 on *Rhizoctonia solani*. Plan. Theory 9:912. doi: 10.3390/plants9070912, PMID: 32707691 PMC7412022

[ref54] Haouhach S. Karkachi N. Oguiba B. Sidaoui A. Chamorro I. Kihal M.. (2020). Three new reports of *Trichoderma* in Algeria: *T. atrobrunneum*, (South) *T. longibrachiatum* (South), and *T. afroharzianum* (Northwest). Microorganisms 8:1455. doi: 10.3390/microorganisms8101455, PMID: 32977378 PMC7597948

[ref55] Harman G. E. (2000). Myths and dogmas of biocontrol changes in perceptions derived from research on *Trichoderma harzinum* T-22. Plant Dis. 84, 377–393. doi: 10.1094/PDIS.2000.84.4.377, PMID: 30841158

[ref56] Harman G. Khadka R. Doni F. Uphoff N. (2021). Benefits to plant health and productivity from enhancing plant microbial symbionts. Front. Plant Sci. 11:610065. doi: 10.3389/fpls.2020.610065, PMID: 33912198 PMC8072474

[ref57] He C. Wang W. Hou J. (2020). Plant performance of enhancing licorice with dual inoculating dark septate endophytes and *Trichoderma viride* mediated via effects on root development. BMC Plant Biol. 20:325. doi: 10.1186/s12870-020-02535-9, PMID: 32646473 PMC7346674

[ref005] Hernández. Cazapal-Monteiro C. F. Arroyo F. L. Silva M. I. Palomero A. M. Paz-Silva A.. (2018). Biological control of soil transmitted helminths (STHs) in a zoological park by using saprophytic fungi. Biol Control, 122, 24–30., PMID: 35281305

[ref58] Herrera-Téllez V. I. Cruz-Olmedo A. K. Plasencia J. Gavilanes-Ruíz M. Arce-Cervantes O. Hernández-León S.. (2019). The protective effect of *Trichoderma asperellum* on tomato plants against *Fusarium oxysporum* and *Botrytis cinerea* diseases involves inhibition of reactive oxygen species production. Int. J. Mol. Sci. 20:2007. doi: 10.3390/ijms20082007, PMID: 31022849 PMC6514666

[ref59] Hinterdobler W. Li G. Spiegel K. Basyouni-Khamis S. Gorfer M. Schmoll M. (2021). *Trichoderma reesei* isolated from Austrian soil with high potential for biotechnological application. Front. Microbiol. 12:552301. doi: 10.3389/fmicb.2021.552301, PMID: 33584603 PMC7876326

[ref60] Illescas M. Morán-Diez M. E. Martínez de Alba Á. E. Hermosa R. Monte E. (2022). Effect of *Trichoderma asperellum* on wheat plants' biochemical and molecular responses, and yield under different water stress conditions. Int. J. Mol. Sci. 23:6782. doi: 10.3390/ijms23126782, PMID: 35743226 PMC9224292

[ref61] Intana W. Kheawleng S. Sunpapao A. (2021). *Trichoderma asperellum* T76-14 released volatile organic compounds against postharvest fruit rot in muskmelons (*Cucumis melo*) caused by *Fusarium incarnatum*. J. Fungi 7:46. doi: 10.3390/jof7010046, PMID: 33445575 PMC7827528

[ref62] Intana W. Wonglom P. Suwannarach N. Sunpapao A. (2022). *Trichoderma asperelloides* PSU-P1 induced expression of pathogenesis-related protein genes against gummy stem blight of muskmelon (*Cucumis melo*) in field evaluation. J. Fungi 8:156. doi: 10.3390/jof8020156, PMID: 35205910 PMC8878962

[ref63] Izquierdo-García L. F. González-Almario A. Cotes A. M. Moreno-Velandia C. A. (2020). *Trichoderma virens* Gl006 and *Bacillus velezensis* Bs006: a compatible interaction controlling *Fusarium* wilt of cape gooseberry. Sci. Rep. 10:6857. doi: 10.1038/s41598-020-63689-y, PMID: 32321998 PMC7176702

[ref64] Jaiswal A. K. Mengiste T. D. Myers J. R. Egel D. S. Hoagland L. A. (2020). Tomato domestication attenuated responsiveness to a beneficial soil microbe for plant growth promotion and induction of systemic resistance to foliar pathogens. Front. Microbiol. 11:604566. doi: 10.3389/fmicb.2020.604566, PMID: 33391227 PMC7775394

[ref65] Ji S. Liu Z. Wang Y. (2021). *Trichoderma*-induced ethylene responsive factor MsERF105 mediates defense responses in *Malus sieversii*. Front. Plant Sci. 12:708010. doi: 10.3389/fpls.2021.708010, PMID: 34777407 PMC8585786

[ref66] Jogaiah S. Abdelrahman M. Tran L. P. Ito S. I. (2018). Different mechanisms of *Trichoderma virens*-mediated resistance in tomato against *Fusarium* wilt involve the jasmonic and salicylic acid pathways. Mol. Plant Pathol. 19, 870–882. doi: 10.1111/mpp.12571, PMID: 28605157 PMC6638079

[ref67] Joo J. H. Hussein K. A. (2022). Biological control and plant growth promotion properties of volatile organic compound-producing antagonistic *Trichoderma* spp. Front. Plant Sci. 13:897668. doi: 10.3389/fpls.2022.897668, PMID: 35958189 PMC9360753

[ref68] Kakabouki I. Tataridas A. Mavroeidis A. Kousta A. Karydogianni S. Zisi C.. (2021). Effect of colonization of *Trichoderma harzianum* on growth development and CBD content of hemp (*Cannabis sativa* L.). Microorganisms 9:518. doi: 10.3390/microorganisms9030518, PMID: 33802427 PMC7998984

[ref69] Kappel L. Kosa N. Gruber S. (2022). The multilateral efficacy of chitosan and *Trichoderma* on sugar beet. J. Fungi 8:137. doi: 10.3390/jof8020137, PMID: 35205892 PMC8879458

[ref70] Kappel L. Münsterkötter M. Sipos G. Escobar Rodriguez C. Gruber S. (2020). Chitin and chitosan remodeling defines vegetative development and *Trichoderma* biocontrol. PLoS Pathog. 16:e1008320. doi: 10.1371/journal.ppat.1008320, PMID: 32078661 PMC7053769

[ref71] Karimi Aghcheh R. Druzhinina I. S. Kubicek C. P. (2013). The putative protein methyltransferase LAE1 of *Trichoderma atroviride* is a key regulator of asexual development and mycoparasitism. PLoS One 8:e67144. doi: 10.1371/journal.pone.0067144, PMID: 23826217 PMC3691206

[ref72] Karuppiah V. Sun J. Li T. Vallikkannu M. Chen J. (2019a). Co-cultivation of *Trichoderma asperellum* GDFS1009 and *Bacillus amyloliquefaciens* 1841 causes differential gene expression and improvement in the wheat growth and biocontrol activity. Front. Microbiol. 10:1068. doi: 10.3389/fmicb.2019.01068, PMID: 31156586 PMC6532653

[ref73] Karuppiah V. Vallikkannu M. Li T. Chen J. (2019b). Simultaneous and sequential based co-fermentations of *Trichoderma asperellum* GDFS1009 and *Bacillus amyloliquefaciens* 1841: a strategy to enhance the gene expression and metabolites to improve the bio-control and plant growth promoting activity. Microb. Cell Fact. 18:185. doi: 10.1186/s12934-019-1233-7, PMID: 31665025 PMC6819339

[ref74] Karuppiah V. Zhixiang L. Liu H. Vallikkannu M. Chen J. (2021). Co-culture of Vel1-over- expressed *Trichoderma asperellum* and *Bacillus amyloliquefaciens*: an eco-friendly strategy to hydrolyze the lignocellulose biomass in soil to enrich the soil fertility, plant growth and disease resistance. Microb. Cell Factories 20:57. doi: 10.1186/s12934-021-01540-3, PMID: 33653343 PMC7927390

[ref75] Khan R. Najeeb S. Mao Z. Ling J. Yang Y. Li Y.. (2020). Bioactive secondary metabolites from *Trichoderma* spp. against phytopathogenic bacteria and root-knot nematode. Microorganisms 8:401. doi: 10.3390/microorganisms8030401, PMID: 32182971 PMC7143365

[ref76] Köhl J. Kolnaar R. Ravensberg W. J. (2019). Mode of action of microbial biological control agents against plant diseases: relevance beyond efficacy. Front. Plant Sci. 10:845. doi: 10.3389/fpls.2019.00845, PMID: 31379891 PMC6658832

[ref77] Kong W. L. Ni H. Wang W. Y. Wu X. Q. (2022). Antifungal effects of volatile organic compounds produced by *Trichoderma koningiopsis* T2 against *Verticillium dahliae*. Front. Microbiol. 13:1013468. doi: 10.3389/fmicb.2022.1013468, PMID: 36212874 PMC9533717

[ref78] Kottb M. Gigolashvili T. Großkinsky D. K. Piechulla B. (2015). *Trichoderma volatiles* effecting Arabidopsis: from inhibition to protection against phytopathogenic fungi. Front. Microbiol. 6:995. doi: 10.3389/fmicb.2015.00995, PMID: 26483761 PMC4586454

[ref79] Kovács C. Csótó A. Pál K. Nagy A. Fekete E. Karaffa L.. (2021). The biocontrol potential of endophytic *Trichoderma* fungi isolated from Hungarian grapevines. Part I. isolation, identification and in vitro studies. Pathogens 10:1612. doi: 10.3390/pathogens10121612, PMID: 34959567 PMC8708432

[ref80] Kubicek C. P. Steindorff A. S. Chenthamara K. Manganiello G. Henrissat B. Zhang J.. (2019). Evolution and comparative genomics of the most common *Trichoderma* species. BMC Genomics 20:485. doi: 10.1186/s12864-019-5680-7, PMID: 31189469 PMC6560777

[ref81] Lamdan N. L. Shalaby S. Ziv T. Kenerley C. M. Horwitz B. A. (2015). Secretome of *Trichoderma* interacting with maize roots: role in induced systemic resistance. Mol. Cell. Proteomics 14, 1054–1063. doi: 10.1074/mcp.M114.046607, PMID: 25681119 PMC4390251

[ref82] Lazazzara V. Vicelli B. Bueschl C. Parich A. Pertot I. Schuhmacher R.. (2021). *Trichoderma* spp. volatile organic compounds protect grapevine plants by activating defense-related processes against downy mildew. Physiol. Plant. 172, 1950–1965. doi: 10.1111/ppl.13406, PMID: 33783004 PMC8360165

[ref83] Leal C. Richet N. Guise J. F. Gramaje D. Armengol J. Fontaine F.. (2021). Cultivar contributes to the beneficial effects of *Bacillus subtilis* PTA-271 and *Trichoderma atroviride* SC1 to protect grapevine against *Neofusicoccum parvum*. Front. Microbiol. 12:726132. doi: 10.3389/fmicb.2021.726132, PMID: 34721323 PMC8552030

[ref84] Li N. Alfiky A. Wang W. Islam M. Nourollahi K. Liu X.. (2018). Volatile compound- mediated recognition and inhibition between *Trichoderma* biocontrol agents and *Fusarium oxysporum*. Front. Microbiol. 9:2614. doi: 10.3389/fmicb.2018.02614, PMID: 30455673 PMC6231246

[ref85] Li X. Leng J. Yu L. Bai H. Li X. Wisniewski M.. (2022). Efficacy of the biocontrol agent *Trichoderma hamatum* against *Lasiodiplodia theobromae* on macadamia. Front. Microbiol. 13:994422. doi: 10.3389/fmicb.2022.994422, PMID: 36118222 PMC9470996

[ref86] Li W. C. Lin T. C. Chen C. L. Liu H. C. Lin H. N. Chao J. L.. (2021). Complete genome sequences and genome-wide characterization of *Trichoderma* biocontrol agents provide new insights into their evolution and variation in genome organization, sexual development, and fungal-plant interactions. Microbiol. Spectr. 9:e0066321. doi: 10.1128/Spectrum.00663-21, PMID: 34908505 PMC8672877

[ref87] Li J. Philp J. Li J. Wei Y. Li H. Yang K.. (2020). *Trichoderma harzianum* inoculation reduces the incidence of Clubroot disease in Chinese cabbage by regulating the rhizosphere microbial community. Microorganisms 8:1325. doi: 10.3390/microorganisms8091325, PMID: 32878079 PMC7563613

[ref88] Li M. Ren Y. He C. Yao J. Wei M. He X. (2022). Complementary effects of dark septate endophytes and *Trichoderma* strains on growth and active ingredient accumulation of *Astragalus mongholicus* under drought stress. J. Fungi 8:920. doi: 10.3390/jof8090920, PMID: 36135646 PMC9506129

[ref89] Liu H. Hao D. Li Y. Wang X. Chen J. (2022). Approaches for the establishment of optimized co-culture system of multiple *Trichoderma* strains for culture metabolites highly effective in cucumber growth promotion. Front. Microbiol. 13:1020077. doi: 10.3389/fmicb.2022.1020077, PMID: 36238592 PMC9551241

[ref90] Liu Y. He P. He P. Munir S. Ahmed A. Wu Y.. (2022). Potential biocontrol efficiency of *Trichoderma* species against oomycete pathogens. Front. Microbiol. 13:974024. doi: 10.3389/fmicb.2022.974024, PMID: 36147847 PMC9487998

[ref91] Lombardi N. Caira S. Troise A. D. Scaloni A. Vitaglione P. Vinale F.. (2020a). *Trichoderma* applications on strawberry plants modulate the physiological processes positively affecting fruit production and quality. Front. Microbiol. 11:1364. doi: 10.3389/fmicb.2020.01364, PMID: 32719661 PMC7350708

[ref92] Lombardi N. Salzano A. M. Troise A. D. Scaloni A. Vitaglione P. Vinale F.. (2020b). Effect of *Trichoderma* bioactive metabolite treatments on the production, quality, and protein profile of strawberry fruits. J. Agric. Food Chem. 68, 7246–7258. doi: 10.1021/acs.jafc.0c01438, PMID: 32426974 PMC8154561

[ref93] López A. C. Alvarenga A. E. Zapata P. D. Luna M. F. Villalba L. L. (2019). *Trichoderma* spp. from Misiones, Argentina: effective fungi to promote plant growth of the regional crop *Ilex paraguariensis* St. Hil. Mycology 10, 210–221. doi: 10.1080/21501203.2019.1606860, PMID: 31632830 PMC6781461

[ref94] Mahmoud G. A. Abdel-Sater M. A. Al-Amery E. Hussein N. A. (2021). Controlling *Alternaria cerealis* MT808477 tomato phytopathogen by *Trichoderma harzianum* and tracking the plant physiological changes. Plan. Theory 10:1846. doi: 10.3390/plants10091846, PMID: 34579379 PMC8470447

[ref95] Malmierca M. G. Cardoza R. E. Alexander N. J. McCormick S. P. Hermosa R. Monte E.. (2012). Involvement of *Trichoderma trichothecenes* in the biocontrol activity and induction of plant defense-related genes. Appl. Environ. Microbiol. 78, 4856–4868. doi: 10.1128/AEM.00385-12, PMID: 22562989 PMC3416386

[ref96] Manganiello G. Nicastro N. Caputo M. Zaccardelli M. Cardi T. Pane C. (2021). Functional hyperspectral imaging by high-related vegetation indices to track the wide-spectrum *Trichoderma* biocontrol activity against soil-borne diseases of baby-leaf vegetables. Front. Plant Sci. 12:630059. doi: 10.3389/fpls.2021.630059, PMID: 33763091 PMC7984460

[ref97] Manganiello G. Sacco A. Ercolano M. R. Vinale F. Lanzuise S. Pascale A.. (2018). Modulation of tomato response to *Rhizoctonia solani* by *Trichoderma harzianum* and its secondary metabolite harzianic acid. Front. Microbiol. 9:1966. doi: 10.3389/fmicb.2018.01966, PMID: 30233507 PMC6127634

[ref98] Manzar N. Kashyap A. S. Goutam R. S. Rajawat M. V. S. Sharma P. K. Sharma S. K.. (2022). *Trichoderma*: advent of versatile biocontrol agent, its secrets and insights into mechanism of biocontrol potential. Sustainability 14:12786. doi: 10.3390/su141912786

[ref99] Marik T. Tyagi C. Balázs D. Urbán P. Szepesi Á. Bakacsy L.. (2019). Structural diversity and bioactivities of peptaibol compounds from the longibrachiatum clade of the filamentous fungal genus *Trichoderma*. Front. Microbiol. 10:1434. doi: 10.3389/fmicb.2019.01434, PMID: 31293557 PMC6606783

[ref100] Marra R. Lombardi N. Piccolo A. Bazghaleh N. Prashar P. Vandenberg A.. (2021). Mineral biofortification and growth stimulation of lentil plants inoculated with *Trichoderma* strains and metabolites. Microorganisms 10:87. doi: 10.3390/microorganisms10010087, PMID: 35056535 PMC8779936

[ref101] Marraschi R. Ferreira A. da Silva Bueno R. N. Leite J. Lucon C. Harakava R.. (2019). A protocol for selection of *Trichoderma* spp. to protect grapevine pruning wounds against *Lasiodiplodia theobromae*. Braz. J. Microbiol. 50, 213–221. doi: 10.1007/s42770-018-0029-y, PMID: 30637650 PMC6863197

[ref102] Martínez-Medina A. Fernández I. Sánchez-Guzmán M. J. Jung S. C. Pascual J. A. Pozo M. J. (2013). Deciphering the hormonal signalling network behind the systemic resistance induced by *Trichoderma harzianum* in tomato. Front. Plant Sci. 4:206. doi: 10.3389/fpls.2013.00206, PMID: 23805146 PMC3690380

[ref103] Martínez-Salgado S. J. Andrade-Hoyos P. Parraguirre Lezama C. Rivera-Tapia A. Luna-Cruz A. Romero-Arenas O. (2021). Biological control of charcoal rot in peanut crop through strains of *Trichoderma* spp., in Puebla, Mexico. Plan. Theory 10:2630. doi: 10.3390/plants10122630, PMID: 34961101 PMC8707606

[ref104] Maruyama C. R. Bilesky-José N. de Lima R. Fraceto L. F. (2020). Encapsulation of *Trichoderma harzianum* preserves enzymatic activity and enhances the potential for biological control. Front. Bioeng. Biotechnol. 8:225. doi: 10.3389/fbioe.2020.00225, PMID: 32269991 PMC7110528

[ref105] Matas-Baca M. Á. Urías García C. Pérez-Álvarez S. Flores-Córdova M. A. Escobedo- Bonilla C. M. Magallanes-Tapia M. A.. (2022). Morphological and molecular characterization of a new autochthonous *Trichoderma* sp. isolate and its biocontrol efficacy against *Alternaria* sp. Saudi. J. Biol. Sci. 29, 2620–2625. doi: 10.1016/j.sjbs.2021.12.052, PMID: 35531149 PMC9072903

[ref106] Mayo S. Gutiérrez S. Malmierca M. G. Lorenzana A. Campelo M. P. Hermosa R.. (2015). Influence of *Rhizoctonia solani* and *Trichoderma* spp. in growth of bean (*Phaseolus vulgaris* L.) and in the induction of plant defense-related genes. Front. Plant Sci. 6:685. doi: 10.3389/fpls.2015.00685, PMID: 26442006 PMC4584982

[ref107] Mayo-Prieto S. Marra R. Vinale F. Rodríguez-González Á. Woo S. L. Lorito M.. (2019). Effect of *Trichoderma velutinum* and *Rhizoctonia solani* on the metabolome of bean plants (*Phaseolus vulgaris* L.). Int. J. Mol. Sci. 20:549. doi: 10.3390/ijms20030549, PMID: 30696057 PMC6387467

[ref108] Mohiddin F. A. Padder S. A. Bhat A. H. Ahanger M. A. Shikari A. B. Wani S. H.. (2021). Phylogeny and optimization of *Trichoderma harzianum* for Chitinase production: evaluation of their antifungal behaviour against the prominent soil borne Phyto-pathogens of temperate India. Microorganisms 9:1962. doi: 10.3390/microorganisms9091962, PMID: 34576858 PMC8471080

[ref109] Monfil V. O. Casas-Flores S. (2014). Molecular mechanisms of biocontrol in *Trichoderma* spp. and their applications in agriculture. Biotechnol. Biol. Trichoderma 8:447. doi: 10.1016/B978-0-444-59576-8.00032-1

[ref110] Moo-Koh F. A. Cristóbal-Alejo J. Andrés M. F. Martín J. Reyes F. Tun-Suárez J. M.. (2022). In vitro assessment of organic and residual fractions of nematicidal culture filtrates from thirteen tropical *Trichoderma* strains and metabolic profiles of most-active. J. Fungi 8:82. doi: 10.3390/jof8010082, PMID: 35050022 PMC8779102

[ref111] Morán-Diez M. E. Carrero-Carrón I. Rubio M. B. Jiménez-Díaz R. M. Monte E. Hermosa R. (2019). Transcriptomic analysis of *Trichoderma atroviride* overgrowing plant-wilting *Verticillium dahliae* reveals the role of a new M14 metallocarboxypeptidase CPA1 in biocontrol. Front. Microbiol. 10:1120. doi: 10.3389/fmicb.2019.01120, PMID: 31191472 PMC6545926

[ref112] Morán-Diez M. E. Tranque E. Bettiol W. Monte E. Hermosa R. (2020). Differential response of tomato plants to the application of three *Trichoderma* species when evaluating the control of *Pseudomonas syringae* populations. Plan. Theory 9:626. doi: 10.3390/plants9050626, PMID: 32422955 PMC7285377

[ref113] Moreno-Ruiz D. Lichius A. Turrà D. Di Pietro A. Zeilinger S. (2020). Chemotropism assays for plant symbiosis and mycoparasitism related compound screening in *Trichoderma atroviride*. Front. Microbiol. 11:601251. doi: 10.3389/fmicb.2020.601251, PMID: 33329491 PMC7729004

[ref114] Mulatu A. Alemu T. Megersa N. Vetukuri R. R. (2021). Optimization of culture conditions and production of bio-fungicides from *Trichoderma* species under solid-state fermentation using mathematical modeling. Microorganisms 9:1675. doi: 10.3390/microorganisms9081675, PMID: 34442753 PMC8400879

[ref115] Naglot A. Goswami S. Rahman I. Shrimali D. D. Yadav K. K. Gupta V. K.. (2015). Antagonistic potential of native *Trichoderma viride* strain against potent tea fungal pathogens in north East India. Plant Pathol. J. 31, 278–289. doi: 10.5423/PPJ.OA.01.2015.0004, PMID: 26361476 PMC4564153

[ref116] Navazio L. Baldan B. Moscatiello R. Zuppini A. Woo S. L. Mariani P.. (2007). Calcium- mediated perception and defense responses activated in plant cells by metabolite mixtures secreted by the biocontrol fungus *Trichoderma atroviride*. BMC Plant Biol. 7:41. doi: 10.1186/1471-2229-7-41, PMID: 17663762 PMC1950503

[ref117] Nawrocka J. Gromek A. Małolepsza U. (2019). Nitric oxide as a beneficial signaling molecule in *Trichoderma atroviride* TRS25- induced systemic defense responses of cucumber plants against *Rhizoctonia solani*. Front. Plant Sci. 10:421. doi: 10.3389/fpls.2019.00421, PMID: 31057564 PMC6478799

[ref118] Nawrocka J. Małolepsza U. Szymczak K. Szczech M. (2018). Involvement of metabolic components, volatile compounds, PR proteins, and mechanical strengthening in multilayer protection of cucumber plants against *Rhizoctonia solani* activated by *Trichoderma atroviride* TRS25. Protoplasma 255, 359–373. doi: 10.1007/s00709-017-1157-1, PMID: 28879466 PMC5756291

[ref119] Ngo M. T. Nguyen M. V. Han J. W. Park M. S. Kim H. Choi G. J. (2021). In vitro and in vivo antifungal activity of *Sorbicillinoids* produced by *Trichoderma longibrachiatum*. J. Fungi 7:428. doi: 10.3390/jof7060428, PMID: 34071658 PMC8229967

[ref120] Nieto-Jacobo M. F. Steyaert J. M. Salazar-Badillo F. B. Nguyen D. V. Rostás M. Braithwaite M.. (2017). Environmental growth conditions of *Trichoderma* spp. affects Indole acetic acid derivatives, volatile organic compounds, and plant growth promotion. Front. Plant Sci. 8:102. doi: 10.3389/fpls.2017.00102, PMID: 28232840 PMC5299017

[ref121] Niu B. Wang W. Yuan Z. Sederoff R. R. Sederoff H. Chiang V. L.. (2020). Microbial interactions within multiple-strain biological control agents impact soil-borne plant disease. Front. Microbiol. 11:585404. doi: 10.3389/fmicb.2020.585404, PMID: 33162962 PMC7581727

[ref122] Nuangmek W. Aiduang W. Kumla J. Lumyong S. Suwannarach N. (2021). Evaluation of a newly identified endophytic fungus, *Trichoderma phayaoense* for plant growth promotion and biological control of gummy stem blight and wilt of muskmelon. Front. Microbiol. 12:634772. doi: 10.3389/fmicb.2021.634772, PMID: 33746927 PMC7973005

[ref123] Organo N. D. Granada S. Pineda H. Sandro J. M. Nguyen V. H. Gummert M. (2022). Assessing the potential of a *Trichoderma*-based compost activator to hasten the decomposition of incorporated rice straw. Sci. Rep. 12:448. doi: 10.1038/s41598-021-03828-1, PMID: 35013411 PMC8748449

[ref124] Oszust K. Cybulska J. Frąc M. (2020). How do *Trichoderma* genus fungi win a nutritional competition battle against soft fruit pathogens? A report on niche overlap nutritional potentiates. Int. J. Mol. Sci. 21:4235. doi: 10.3390/ijms21124235, PMID: 32545883 PMC7352470

[ref125] Panchalingam H. Powell D. Adra C. Foster K. Tomlin R. Quigley B. L.. (2022). Assessing the various antagonistic mechanisms of *Trichoderma* strains against the brown root rot pathogen *Pyrrhoderma noxium* infecting heritage fig trees. J. Fungi 8:1105. doi: 10.3390/jof8101105, PMID: 36294670 PMC9605450

[ref126] Park Y. H. Chandra Mishra R. Yoon S. Kim H. Park C. Seo S. T.. (2019). Endophytic *Trichoderma citrinoviride* isolated from mountain-cultivated ginseng (*Panax ginseng*) has great potential as a biocontrol agent against ginseng pathogens. J. Ginseng Res. 43, 408–420. doi: 10.1016/j.jgr.2018.03.002, PMID: 31308813 PMC6606899

[ref127] Pescador L. Fernandez I. Pozo M. J. Romero-Puertas M. C. Pieterse C. Martínez- Medina A. (2022). Nitric oxide signalling in roots is required for MYB72-dependent systemic resistance induced by *Trichoderma* volatile compounds in *Arabidopsis*. J. Exp. Bot. 73, 584–595. doi: 10.1093/jxb/erab294, PMID: 34131708 PMC8757496

[ref128] Phoka N. Suwannarach N. Lumyong S. Ito S. I. Matsui K. Arikit S.. (2020). Role of volatiles from the endophytic fungus *Trichoderma asperelloides* PSU-P1 in biocontrol potential and in promoting the plant growth of *Arabidopsis thaliana*. J. Fungi 6:341. doi: 10.3390/jof6040341, PMID: 33291279 PMC7762097

[ref129] Pocurull M. Fullana A. M. Ferro M. Valero P. Escudero N. Saus E.. (2020). Commercial formulates of *Trichoderma* induce systemic plant resistance to *Meloidogyne incognita* in tomato and the effect is additive to that of the Mi-1.2 resistance gene. Front. Microbiol. 10:3042. doi: 10.3389/fmicb.2019.03042, PMID: 32076417 PMC7006539

[ref130] Pollard-Flamand J. Boulé J. Hart M. Úrbez-Torres J. R. (2022). Biocontrol activity of *Trichoderma* species isolated from grapevines in British Columbia against botryosphaeria dieback fungal pathogens. J. Fungi 8:409. doi: 10.3390/jof8040409, PMID: 35448640 PMC9030288

[ref131] Poveda J. Abril-Urias P. Escobar C. (2020). Biological control of plant-parasitic nematodes by filamentous fungi inducers of resistance: *Trichoderma*, Mycorrhizal and Endophytic Fungi. Front. Microbiol. 11:992. doi: 10.3389/fmicb.2020.00992, PMID: 32523567 PMC7261880

[ref132] Poveda J. Hermosa R. Monte E. Nicolás C. (2019). *Trichoderma harzianum* favours the access of *arbuscular mycorrhizal* fungi to non-host Brassicaceae roots and increases plant productivity. Sci. Rep. 9:11650. doi: 10.1038/s41598-019-48269-z, PMID: 31406170 PMC6690897

[ref133] Rao Y. Zeng L. Jiang H. Mei L. Wang Y. (2022). *Trichoderma atroviride* LZ42 releases volatile organic compounds promoting plant growth and suppressing Fusarium wilt disease in tomato seedlings. BMC Microbiol. 22:88. doi: 10.1186/s12866-022-02511-3, PMID: 35382732 PMC8981656

[ref134] Rashmi S. Maurya S. Upadhyay R. S. (2016). The improvement of competitive saprophytic capabilities of *Trichoderma* species through the use of chemical mutagens. Braz. J. Microbiol. 47, 10–17. doi: 10.1016/j.bjm.2015.11.003, PMID: 26887221 PMC4822768

[ref135] Rawal R. Scheerens J. C. Fenstemaker S. M. Francis D. M. Miller S. A. Benitez M. S. (2022). Novel *Trichoderma* isolates alleviate water deficit stress in susceptible tomato genotypes. Front. Plant Sci. 13:869090. doi: 10.3389/fpls.2022.869090, PMID: 35586213 PMC9108677

[ref136] Rees H. J. Drakulic J. Cromey M. G. Bailey A. M. Foster G. D. (2022). Endophytic *Trichoderma* spp. can protect strawberry and privet plants from infection by the fungus *Armillaria mellea*. PLoS One 17:e0271622. doi: 10.1371/journal.pone.0271622, PMID: 35913938 PMC9342734

[ref137] Risoli S. Cotrozzi L. Sarrocco S. Nuzzaci M. Pellegrini E. Vitti A. (2022). *Trichoderma*-induced resistance to *Botrytis cinerea* in *Solanum* species: a meta-analysis. Plan. Theory 11:180. doi: 10.3390/plants11020180, PMID: 35050068 PMC8780288

[ref138] Ruangwong O. U. Pornsuriya C. Pitija K. Sunpapao A. (2021a). Biocontrol mechanisms of *Trichoderma koningiopsis* PSU3-2 against postharvest anthracnose of chili pepper. J. Fungi 7:276. doi: 10.3390/jof7040276, PMID: 33916921 PMC8067587

[ref139] Ruangwong O. U. Wonglom P. Suwannarach N. Kumla J. Thaochan N. Chomnunti P.. (2021b). Volatile organic compound from *Trichoderma asperelloides* TSU1: impact on plant pathogenic fungi. J. Fungi 7:187. doi: 10.3390/jof7030187, PMID: 33807949 PMC7999943

[ref140] Rubio M. B. Quijada N. M. Pérez E. Domínguez S. Monte E. Hermosa R. (2014). Identifying beneficial qualities of *Trichoderma parareesei* for plants. Appl. Environ. Microbiol. 80, 1864–1873. doi: 10.1128/AEM.03375-13, PMID: 24413597 PMC3957631

[ref141] Samuelian S. (2016). Potential of *Trichoderma harzianum* for control of banana leaf fungal pathogens when applied with a food source and an organic adjuvant. 3 Biotech 6:8. doi: 10.1007/s13205-015-0327-0, PMID: 28330078 PMC4701706

[ref142] Samuels G. J. Dodd S. L. Lu B. S. Petrini O. Schroers H. J. Druzhinina I. S. (2006). The *Trichoderma koningii* aggregate species. Stud. Mycol. 56, 67–133. doi: 10.3114/sim.2006.56.03, PMID: 18490990 PMC2104733

[ref143] Sánchez-Montesinos B. Santos M. Moreno-Gavíra A. Marín-Rodulfo T. Gea F. J. Diánez F. (2021). Biological control of fungal diseases by *Trichoderma aggressivum* f. *europaeum* and its compatibility with fungicides. J. Fungi 7:598. doi: 10.3390/jof7080598, PMID: 34436137 PMC8397002

[ref144] Santos M. Santos L. Costa D. Vieira T. A. Lustosa D. C. (2020). *Trichoderma* spp. on treatment of *Handroanthus serratifolius* seeds: effect on seedling germination and development. Heliyon 6:e04044. doi: 10.1016/j.heliyon.2020.e04044, PMID: 32518852 PMC7270539

[ref145] Saravanakumar K. Fan L. Fu K. Yu C. Wang M. Xia H.. (2016). Cellulase from *Trichoderma harzianum* interacts with roots and triggers induced systemic resistance to foliar disease in maize. Sci. Rep. 6:35543. doi: 10.1038/srep35543, PMID: 27830829 PMC5103226

[ref146] Saravanakumar K. Li Y. Yu C. Wang Q. Q. Wang M. Sun J.. (2017). Effect of *Trichoderma harzianum* on maize rhizosphere microbiome and biocontrol of Fusarium stalk rot. Sci. Rep. 7:1771. doi: 10.1038/s41598-017-01680-w, PMID: 28496167 PMC5431858

[ref147] Şesan T. E. Oancea A. O. Ştefan L. M. Mănoiu V. S. Ghiurea M. Răut I.. (2020). Effects of foliar treatment with a *Trichoderma* plant biostimulant consortium on *Passiflora caerulea* L. yield and quality. Microorganisms 8:123. doi: 10.3390/microorganisms8010123, PMID: 31963272 PMC7023023

[ref148] Shaw S. Le Cocq K. Paszkiewicz K. Moore K. Winsbury R. de Torres Zabala M.. (2016). Transcriptional reprogramming underpins enhanced plant growth promotion by the biocontrol fungus *Trichoderma hamatum* GD12 during antagonistic interactions with *Sclerotinia sclerotiorum* in soil. Mol. Plant Pathol. 17, 1425–1441. doi: 10.1111/mpp.12429, PMID: 27187266 PMC6638342

[ref149] Shobha B. Lakshmeesha T. R. Ansari M. A. Almatroudi A. Alzohairy M. A. Basavaraju S.. (2020). Mycosynthesis of ZnO nanoparticles using *Trichoderma* spp. isolated from rhizosphere soils and its synergistic antibacterial effect against *Xanthomonas oryzae* pv. *oryzae*. J. Fungi 6:181. doi: 10.3390/jof6030181, PMID: 32962271 PMC7558757

[ref150] Shoresh M. Harman G. E. (2010). Differential expression of maize chitinases in the presence or absence of *Trichoderma harzianum* strain T22 and indications of a novel exo-endo-heterodimeric chitinase activity. BMC Plant Biol. 10, 136–111. doi: 10.1186/1471-2229-10-136 20594307 PMC3017806

[ref151] Singh B. N. Dwivedi P. Sarma B. K. Singh G. S. Singh H. B. (2019). A novel function of N-signaling in plants with special reference to *Trichoderma* interaction influencing plant growth, nitrogen use efficiency, and cross talk with plant hormones. 3 Biotech 9:109. doi: 10.1007/s13205-019-1638-3, PMID: 30863693 PMC6393646

[ref152] Sokhandani Z. Moosavi M. R. Basirnia T. (2016). Optimum concentrations of *Trichoderma longibrachiatum* and cadusafos for controlling *Meloidogyne javanica* on Zucchini plants. J. Nematol. 48, 54–63. doi: 10.21307/jofnem-2017-009, PMID: 27168653 PMC4859618

[ref153] Stracquadanio C. Quiles J. M. Meca G. Cacciola S. O. (2020). Antifungal activity of bioactive metabolites produced by *Trichoderma asperellum* and *Trichoderma atroviride* in liquid medium. J. Fungi 6:263. doi: 10.3390/jof6040263, PMID: 33139651 PMC7712451

[ref154] Sui L. Li J. Philp J. Yang K. Wei Y. Li H.. (2022). *Trichoderma atroviride* seed dressing influenced the fungal community and pathogenic fungi in the wheat rhizosphere. Sci. Rep. 12:9677. doi: 10.1038/s41598-022-13669-1, PMID: 35690652 PMC9188553

[ref155] Sun J. Karuppiah V. Li Y. Pandian S. Kumaran S. Chen J. (2022). Role of cytochrome P450 genes of *Trichoderma atroviride* T23 on the resistance and degradation of dichlorvos. Chemosphere 290:133173. doi: 10.1016/j.chemosphere.2021.133173, PMID: 34914953

[ref156] Sun R. Y. Liu Z. C. Fu K. Fan L. Chen J. (2012). *Trichoderma* biodiversity in China. J. Appl. Genet. 53, 343–354. doi: 10.1007/s13353-012-0093-1, PMID: 22528042

[ref157] Swain H. Adak T. Mukherjee A. K. Sarangi S. Samal P. Khandual A.. (2021). Seed biopriming with *Trichoderma* strains isolated from tree bark improves plant growth, antioxidative defense system in Rice and enhance straw degradation capacity. Front. Microbiol. 12:633881. doi: 10.3389/fmicb.2021.633881, PMID: 33717027 PMC7952651

[ref158] Tamizi A. A. Mat-Amin N. Weaver J. A. Olumakaiye R. T. Akbar M. A. Jin S.. (2022). Genome sequencing and analysis of *Trichoderma* (Hypocreaceae) isolates exhibiting antagonistic activity against the papaya dieback pathogen, *Erwinia mallotivora*. J. Fungi 8:246. doi: 10.3390/jof8030246, PMID: 35330248 PMC8949440

[ref159] Thambugala K. M. Daranagama D. A. Phillips A. Kannangara S. D. Promputtha I. (2020). Fungi vs. fungi in biocontrol: an overview of fungal antagonists applied against fungal plant pathogens. Front. Cell. Infect. Microbiol. 10:604923. doi: 10.3389/fcimb.2020.604923, PMID: 33330142 PMC7734056

[ref160] Tian Y. Tan Y. Liu N. Yan Z. Liao Y. Chen J.. (2016). Detoxification of deoxynivalenol via glycosylation represents novel insights on antagonistic activities of *Trichoderma* when confronted with *Fusarium graminearum*. Toxins 8:335. doi: 10.3390/toxins8110335, PMID: 27854265 PMC5127131

[ref161] Tian Y. Tan Y. Yan Z. Liao Y. Chen J. De Boevre M.. (2018). Antagonistic and detoxification potentials of *Trichoderma* isolates for control of zearalenone (ZEN) producing *Fusarium graminearum*. Front. Microbiol. 8:2710. doi: 10.3389/fmicb.2017.02710, PMID: 29403455 PMC5778118

[ref162] Tilocca B. Cao A. Migheli Q. (2020). Scent of a killer: microbial volatilome and its role in the biological control of plant pathogens. Front. Microbiol. 11:41. doi: 10.3389/fmicb.2020.00041, PMID: 32117096 PMC7018762

[ref163] Tseng Y. H. Rouina H. Groten K. Rajani P. Furch A. Reichelt M.. (2020). An endophytic *Trichoderma* strain promotes growth of its hosts and defends against pathogen attack. Front. Plant Sci. 11:573670. doi: 10.3389/fpls.2020.573670, PMID: 33424876 PMC7793846

[ref164] Tyśkiewicz R. Nowak A. Ozimek E. Jaroszuk-Ściseł J. (2022). *Trichoderma*: the current status of its application in agriculture for the biocontrol of fungal phytopathogens and stimulation of plant growth. Int. J. Mol. Sci. 23:2329. doi: 10.3390/ijms23042329, PMID: 35216444 PMC8875981

[ref165] Velasco P. Rodríguez V. M. Soengas P. Poveda J. (2021). *Trichoderma hamatum* increases productivity, glucosinolate content and antioxidant potential of different leafy *brassica* vegetables. Plan. Theory 10:2449. doi: 10.3390/plants10112449, PMID: 34834812 PMC8619120

[ref166] Vicente I. Baroncelli R. Morán-Diez M. E. Bernardi R. Puntoni G. Hermosa R.. (2020). Combined comparative genomics and gene expression analyses provide insights into the terpene synthases inventory in *Trichoderma*. Microorganisms 8:1603. doi: 10.3390/microorganisms8101603, PMID: 33081019 PMC7603203

[ref167] Viriyasuthee W. Jogloy S. Saksirirat W. Saepaisan S. Gleason M. L. Chen R. S. (2019). Biological control of Alternaria leaf spot caused by *Alternaria* spp. in Jerusalem artichoke (*Helianthus tuberosus* L.) under two fertilization regimes. Plan. Theory 8:463. doi: 10.3390/plants8110463, PMID: 31671613 PMC6918389

[ref168] Viterbo A. Harel M. Horwitz B. A. Chet I. Mukherjee P. K. (2005). *Trichoderma* mitogen-activated protein kinase signaling is involved in induction of plant systemic resistance. Appl. Environ. Microbiol. 71, 6241–6246. doi: 10.1128/AEM.71.10.6241-6246.2005, PMID: 16204544 PMC1266020

[ref169] Vos C. M. De Cremer K. Cammue B. P. De Coninck B. (2015). The toolbox of *Trichoderma* spp. in the biocontrol of *Botrytis cinerea* disease. Mol. Plant Pathol. 16, 400–412. doi: 10.1111/mpp.12189, PMID: 25171761 PMC6638538

[ref170] Wang Y. Chen H. Ma L. Gong M. Wu Y. Bao D.. (2022). Use of CRISPR-Cas tools to engineer *Trichoderma* species. Microb. Biotechnol. 15, 2521–2532. doi: 10.1111/1751-7915.14126, PMID: 35908288 PMC9518982

[ref171] Wang R. Liu C. Jiang X. Tan Z. Li H. Xu S.. (2022). The newly identified *Trichoderma harzianum* partitivirus (ThPV2) does not diminish spore production and biocontrol activity of its host. Viruses 14:1532. doi: 10.3390/v14071532, PMID: 35891512 PMC9317543

[ref172] Wang H. Zhang R. Duan Y. Jiang W. Chen X. Shen X.. (2021). The endophytic strain *Trichoderma asperellum* 6S-2: an efficient biocontrol agent against apple replant disease in China and a potential plant-growth-promoting fungus. J. Fungi 7:1050. doi: 10.3390/jof7121050, PMID: 34947033 PMC8705406

[ref173] Wang H. Zhang R. Mao Y. Jiang W. Chen X. Shen X.. (2022). Effects of *Trichoderma asperellum* 6S-2 on apple tree growth and replanted soil microbial environment. J. Fungi 8:63. doi: 10.3390/jof8010063, PMID: 35050003 PMC8778220

[ref174] Xu H. Yan L. Zhang M. Chang X. Zhu D. Wei D.. (2022). Changes in the density and composition of rhizosphere pathogenic *Fusarium* and beneficial *Trichoderma* contributing to reduced root rot of intercropped soybean. Pathogens 11:478. doi: 10.3390/pathogens11040478, PMID: 35456153 PMC9031213

[ref175] Ye L. Zhao X. Bao E. Li J. Zou Z. Cao K. (2020). Bio-organic fertilizer with reduced rates of chemical fertilization improves soil fertility and enhances tomato yield and quality. Sci. Rep. 10:177. doi: 10.1038/s41598-019-56954-2, PMID: 31932626 PMC6957517

[ref176] You J. Li G. Li C. Zhu L. Yang H. Song R.. (2022). Biological control and plant growth promotion by volatile organic compounds of *Trichoderma koningiopsis* T-51. J. Fungi 8:131. doi: 10.3390/jof8020131, PMID: 35205885 PMC8875031

[ref177] Yuan M. Huang Y. Ge W. Jia Z. Song S. Zhang L.. (2019). Involvement of jasmonic acid, ethylene and salicylic acid signaling pathways behind the systemic resistance induced by *Trichoderma longibrachiatum* H9 in cucumber. BMC Genomics 20:144. doi: 10.1186/s12864-019-5513-8, PMID: 30777003 PMC6379975

[ref178] Zaid R. Koren R. Kligun E. Gupta R. Leibman-Markus M. Mukherjee P. K.. (2022). Gliotoxin, an immunosuppressive fungal metabolite, primes plant immunity: evidence from *Trichoderma virens*-tomato interaction. MBio 13:e0038922. doi: 10.1128/mbio.00389-22, PMID: 35862794 PMC9426506

[ref179] Zhang J. Chen J. Liu Z. Guan S. Li J. Zhang C.. (2017). Evaluation of the combined use of *Trichoderma atroviride* metabolite and brassinolide in the promotion of vegetable growth and control of *Botrytis cinere*. J. Shanghai Jiaotong Univ. 35, 1–7. doi: 10.3969/J.ISSN.1671-9964.2017.05.001

[ref008] Zhang S. Gan Y. Liu J. Zhou J. Xu B. (2020). Optimization of the fermentation media and parameters for the bio-control potential of *Trichoderma longibrachiatum* T6 against nematodes. Front Microbiol. 11:574601., PMID: 33101249 10.3389/fmicb.2020.574601 PMC7554348

[ref180] Zhang F. Huo Y. Cobb A. B. Luo G. Zhou J. Yang G.. (2018). *Trichoderma* biofertilizer links to altered soil chemistry, altered microbial communities, and improved grassland biomass. Front. Microbiol. 9:848. doi: 10.3389/fmicb.2018.00848, PMID: 29760689 PMC5937142

[ref181] Zhang C. Wang W. Hu Y. Peng Z. Ren S. Xue M.. (2022). A novel salt-tolerant strain *Trichoderma atroviride* HN082102.1 isolated from marine habitat alleviates salt stress and diminishes cucumber root rot caused by *Fusarium oxysporum*. BMC Microbiol. 22:67. doi: 10.1186/s12866-022-02479-0, PMID: 35232373 PMC8887007

[ref182] Zhang C. Wang W. Xue M. Liu Z. Zhang Q. Hou J.. (2021). The combination of a biocontrol agent *Trichoderma asperellum* SC012 and hymexazol reduces the effective fungicide dose to control *Fusarium* wilt in cowpea. J. Fungi 7:685. doi: 10.3390/jof7090685, PMID: 34575723 PMC8471890

[ref183] Zhang Y. Xiao J. Yang K. Wang Y. Tian Y. Liang Z. (2022). Transcriptomic and metabonomic insights into the biocontrol mechanism of *Trichoderma asperellum* M45a against watermelon *Fusarium* wilt. PLoS One 17:e0272702. doi: 10.1371/journal.pone.0272702, PMID: 35947630 PMC9365129

[ref184] Zheng H. Qiao M. Lv Y. Du X. Zhang K. Q. Yu Z. (2021). New species of *Trichoderma* isolated as endophytes and saprobes from Southwest China. J. Fungi 7:467. doi: 10.3390/jof7060467, PMID: 34207925 PMC8230185

[ref004] Zhou D. Huang X. F. Guo J. Dos-Santos M. L. Vivanco. (2018). *Trichoderma gamsii* affected herbivore feeding behaviour on arabidopsis thaliana by modifying the leaf metabolome and phytohormones. Microb Biotechnol. 11, 1195–1206., PMID: 30221488 10.1111/1751-7915.13310 PMC6196387

[ref185] Zhu N. Zhou J. J. Zhang S. W. Xu B. L. (2022). Mechanisms of *Trichoderma longibrachiatum* T6 fermentation against *Valsa mali* through inhibiting its growth and reproduction, pathogenicity and gene expression. J. Fungi 8:113. doi: 10.3390/jof8020113, PMID: 35205867 PMC8875883

